# Investigating the epidemiological and economic effects of a third-party certification policy for restaurants with COVID-19 prevention measures

**DOI:** 10.1038/s41598-023-34498-w

**Published:** 2023-05-11

**Authors:** Kazuya Hirokawa, Jumpei Hirota, Daiji Kawaguchi, Yusuke Masaki, Chiaki Onita

**Affiliations:** 1 Graduate School of Public Policy, The University of Tokyo, 7-3-1 Hongo, Bunkyo-ku, Tokyo, 113-8656 Japan; 2 RIETI, Tokyo, Japan; 3 IZA, Bonn, Germany

**Keywords:** Health policy, Public health, Infectious diseases

## Abstract

This study investigates the effects of a third-party certification policy for restaurants (including bars) that comply with indoor infection-prevention measures on COVID-19 cases and economic activities. We focus on the case of Yamanashi Prefecture in Japan, which introduced a third-party certification policy that accredits facilities, predominantly restaurants, that comply with the designated guidelines. We employ a difference-in-differences design for each of our epidemiological and economic analyses. The estimation results show that, from July 2020 to April 2021, the certification policy reduced the total number of new infection cases by approximately 45.3% (848 cases), while increasing total sales and the number of customers per restaurant by approximately 12.8% (3.21 million Japanese yen or $30,000) and 30.3% (2909 customers), respectively, compared to the non-intervention scenarios. The results suggest that a third-party certification policy can be an effective policy to mitigate the trade-off between economic activities and infection prevention during a pandemic, especially when effective vaccines are not widely available.

## Introduction

During the COVID-19 pandemic, national and regional governments introduced different non-pharmaceutical interventions to prevent its spread. In line with this policy trend, several papers have reviewed the effectiveness of various non-pharmaceutical interventions in controlling infections^1–4^. Interventions that have been studied by econometric methods include lockdowns (including people voluntarily refraining from going out), school closures, social distancing, mask mandates, and bans on large gatherings^5–8^. Along with these policies, governments have also imposed regulations on restaurants, such as operating restrictions, if not completely shutting them down, on the grounds that eating and drinking indoors are associated with high risks of COVID-19 transmission, as indicated by studies analyzing the routes of infection among infected persons and droplet and airborne transmission in restaurants^9–12^.

In response to the risk of infection in restaurants, governments have implemented policies to restrict business operations when infections spread and to reopen the economy when infections decline, but these policies often suffer from a trade–off between managing infections and maintaining the economy^13^. Regulatory policies that involve shutting down or shortening operation hours have been reported to effectively reduce the number of infected people in the early stages of the COVID-19 pandemic^14,15^. Nevertheless, these intensive measures, while generating positive impacts on food retailers ^16^, incurred extensive economic losses for restaurants^17^, thus becoming a major source of concern for restaurant entrepreneurs ^18^. Therefore, when the infection situation settles down for a moment, governments switch to encourage restaurants to reopen for business through policies such as distribution of subsidies. Indeed, subsidizing policies are reported to have increased customer footfall and recruitment in food & service sectors^19^. Re-opening strategies, however, also face challenges. The recovery rate of human mobility is relatively small compared with the significant declines during the lockdown phase, because consumers are cautious about the infection risks of dining out^20,21^. Furthermore, resuming the economy has often led to a resurgence of the COVID-19 contagion and repeated regulations^22–24^. As a result of the policy environment that has seesawed between regulation and deregulation, food and beverage (F&B) industries has suffered unprecedentedly significant financial damage and job losses throughout the world^25–28^.

Thus, policymakers have faced growing needs for measures that balance infection control and economic sustainability in restaurants, though, to the best of our knowledge, none of the previous studies have examined the policy effects from these two perspectives simultaneously. For this reason, we will individually review the two policy effects, epidemiological and economic, on restaurants to summarize the current state of what has already been found useful for both infection control and business maintenance in restaurants. First, for the infection-mitigating measures, inhibiting close contact and aerosol infection has been recognized to be effective. Some studies have conducted computer simulations and analyzed the relationship between seating position and airflow during a cluster outbreak. They revealed that the risk of infection by short- and long-range airborne routes is high, compared to the risk of infection by fomite, making it effective to wear masks, increase seating distance, install partitions, and improve ventilation facilities^11,29–33^. Other studies have deployed natural experiments by using data aggregated by geographical units (e.g., county, city) and concluded that mask-wearing mandates and capacity limits can control the infection growth rate even after reopening restaurants^34–36^. In particular, researchers have found that limiting indoor capacity is highly effective, with regulations to shorten operation hours not significantly reducing the number of infection cases when the capacity measures have already been implemented^37^.

From an economic perspective, conversion to different business forms has been shown to be effective. For example, many studies have shown that, rather than offering discounts, shifting to contactless forms of sales, such as take-out and drone delivery, had a positive impact on sales or customer impressions^38–40^. Furthermore, studies based on questionnaires have indicated that consumers consider hygiene (e.g., employees wearing masks, disinfection, ventilation, etc.) as a major selection criterion when choosing a restaurant amid infection risks^41–47^.

These studies suggest that infection-control measures in food and beverage outlets may be effective in controlling infection and maintaining the economy. None of them, however, provides direct evidence of policies on the two outcomes at the same time, prohibiting us from assessing the compatibility of the policies. We aim to fill this gap by assessing whether a third-party COVID-19 safety certification policy for restaurants can overcome the trade-off between infection prevention and business operations. The purpose of the certification is to ensure that restaurants comply with comprehensive guidelines for infection control, thereby both maintaining business and preventing the spread of infection. To the authors’ knowledge, a third-party certification policy for in-store infection control measures is implemented only in Japan and a few other countries in the world, such as Singapore^48^. In this study, we will examine the case of Yamanashi Prefecture, the first prefecture in Japan to promote such certification.

In May 2020, Yamanashi Prefecture introduced the Yamanashi Green Zone Certification (GZ), a third-party certification policy for infection-prevention measures. The purpose of this policy is to allow consumers to patronize businesses with peace of mind by officially certifying restaurants (including bars), hotels, wineries, breweries, and other businesses that comply with infection-control guidelines^49,50^. Certification is conditional on passing a rigorous on-site inspection. Once a violation is found, the certification may be revoked. Certified businesses are generally exempted from the prefectural government’s requests to close or shorten hours. In the unlikely event that such a request is necessary, they are given priority in receiving financial assistance. The number of GZ-certified restaurants has increased gradually since the first approvals on July 17, 2020. As of April 30, 2021, more than 4000 restaurants in Yamanashi were certified, and the acquisition rate was around 96%^51^ (see Supplementary Information Fig. C.2).

Many prefectures introduced similar policies later, but they were not as strict as that of Yamanashi. For example, some prefectures, including Tokyo, have provided stickers to businesses that declared compliance with infection-prevention guidelines, but the accreditation only required self-reporting documents^52^. Other prefectures, such as Gunma and Tottori, introduced a certification policy with both accreditation requirements and introduction timing similar to the GZ certification policy, but their area-wide accreditation rates were considerably low (i.e., around 20% for Gunma as of October 2021^53^) (see Supplementary Information E.2).

**Figure 1:**
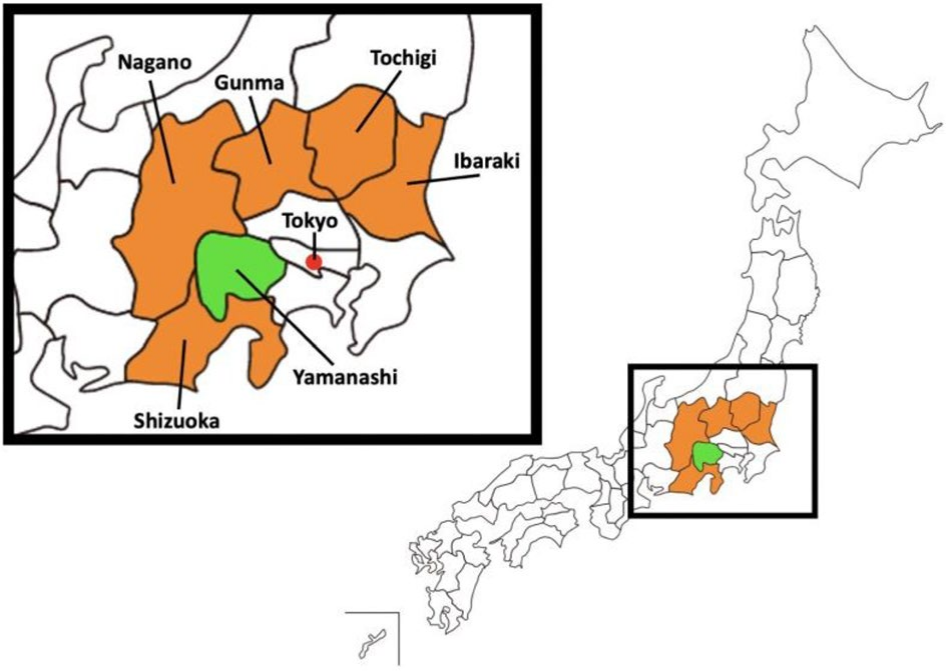
Location of treatment and control prefectures. Treatment (Yamanashi) and control (Shizuoka, Nagano, Gunma, Tochigi, and Ibaraki) prefectures are in green and orange, respectively. Control prefectures were selected because their population density and distance from Tokyo are similar to those of the treatment prefecture.

**Figure 2:**
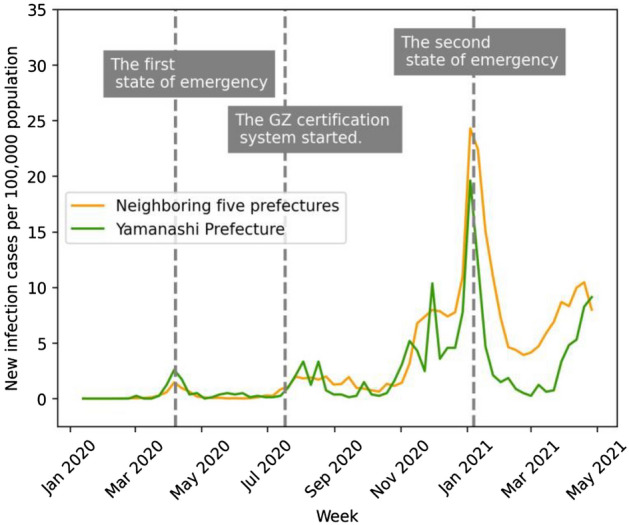
Time series of new infection cases by week. The vertical axis is the new infection cases per 100,000 population per week. The green line is the trend in Yamanashi Prefecture, while the orange line is the average trend of five neighboring prefectures. The gray dotted lines signify the onset of the first and second state of emergency declarations in Tokyo.

**Figure 3:**
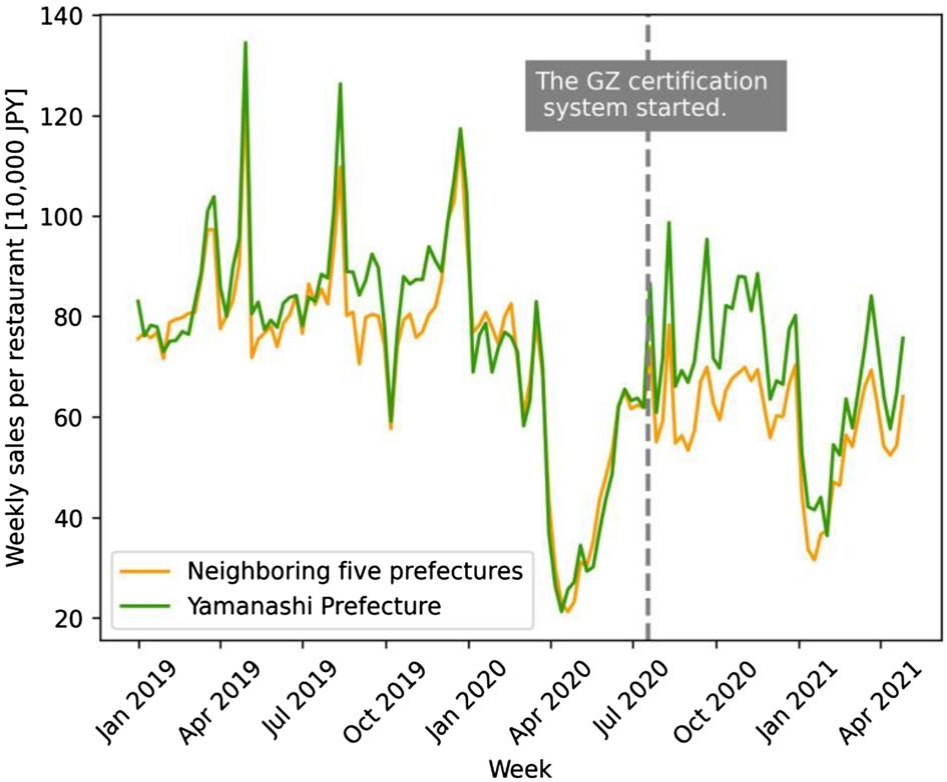
Time series of weekly sales per restaurant in Yamanashi Prefecture and neighboring five prefectures. The vertical axis shows the weekly sales per restaurant (JPY 1 million) made through the cloud POS register “Postas” provided by Postas Corporation. The green line is the trend in Yamanashi Prefecture, while the orange line is the average trend of five neighboring prefectures. The gray dotted line signifies the week when the GZ certification policy approved the first group of restaurants.

**Figure 4:**
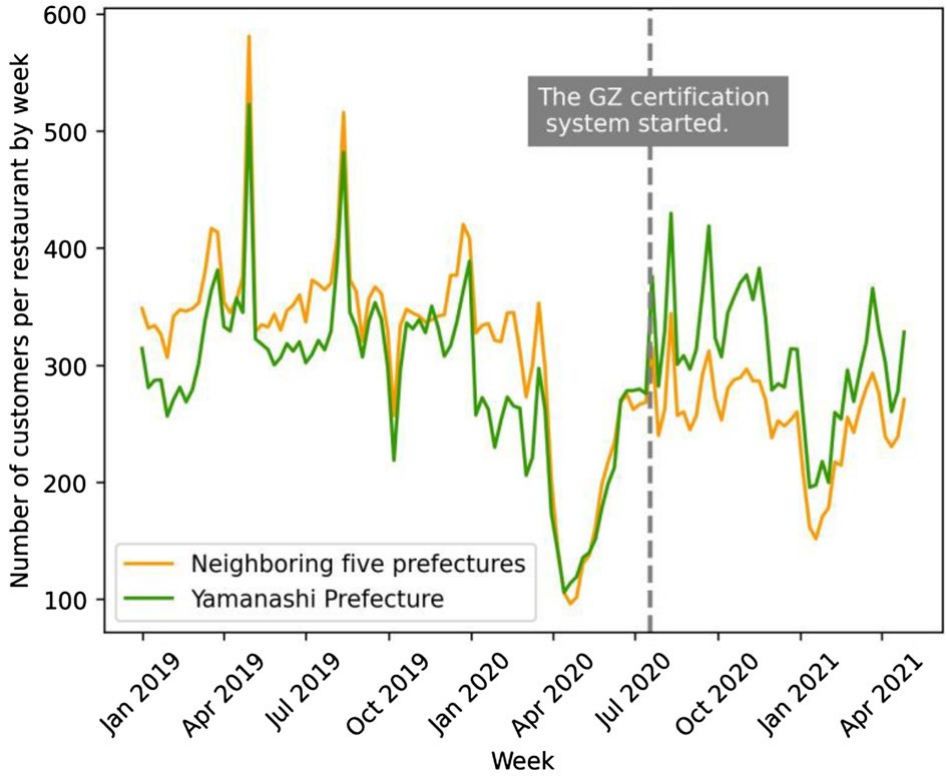
Time series of the number of customers per restaurant by week in Yamanashi Prefecture and neighboring five prefectures. The vertical axis is the weekly number of customers per restaurant recorded through the “Postas” registration system. The green line is the trend in Yamanashi Prefecture, while the orange line is the average trend of five neighboring prefectures. The gray dotted line signifies the week when the GZ certification policy approved the first group of restaurants.

We hypothesize that the GZ certification policy had positive effects on both decreasing the number of new infection cases and increasing restaurant revenues in Yamanashi Prefecture during the COVID-19 pandemic. This expectation is based on first-hand evidence that infection cases and economic damage to restaurants in the prefecture seemed to be under control compared to neighboring prefectures after the introduction of the certification policy (see Fig. 1 for the geographical location and Figs. 2, 3, and 4 for time-series graphs).

In our study, we separately evaluate the epidemiological and economic effects of the GZ certification policy by using a difference-in-differences design, exploiting the incremental introduction of the certification. We compare Yamanashi Prefecture, the treatment group, with the control group of five neighboring prefectures. Selection of the control group is based on similarity of population density and proximity to Tokyo, the epicenter of COVID-19, to remove endogeneity. The epidemiological model analyzes the effect of an increase in the number of certified restaurants on the rate of change in the number of new COVID-19 cases. Incorporating the susceptible-infectious-recovered (SIR) model^54^, our model captures the number of both susceptible and infected populations in a given time period to account for the area’s infection status. To evaluate the economic effect, we analyze the effect of an increase in the number of certified restaurants on the average daily restaurant point-of-sale (POS) transactions and customers in the prefecture. The results indicate that the third-party certification was effective in reducing the number of new infections while simultaneously increasing restaurant sales and customer traffic. This finding has a practical implication that infection-prevention measures in high-risk of virus exposure can deter infection spread and revitalize economic activity.

The contribution of this study is twofold. First, our study is the first paper to assess a policy aiming to overcome the trade-off between preventing new infections and maintaining economic activity. Second, we employ a natural experiment, rather than a computer simulation, and construct an estimation model that removes endogeneity, which prior studies have not addressed. Our SIR-model-based equation successfully captures the dynamic of COVID-19 contagion and thereby can detect policy impacts with less measurement error.

## Methods

This section describes the main datasets, estimation equations, and treatment and control groups.

### Main datasets

In this section, data for the main explanatory and dependent variables are described. Data for the control variables are explained in the Supplementary Information.

#### Green zone certification

The explanatory variable is the cumulative number of GZ-certified restaurants. The certification policy targets various types of facilities, such as restaurants, hotels, breweries, and wineries. In this paper, we focused on restaurants, which are deemed to be the main route of infection^9^. The dataset of individual establishments’ GZ certification acquisition dates was provided by the department in charge of the certification policy at the Yamanashi Prefectural Office.

#### COVID-19 cases

One of the dependent variables is the number of daily new COVID-19 cases in each prefecture. The data were obtained from the Japanese Broadcasting Corporation’s (NHK) “Special site: New coronaviruses—Number of cases by prefecture”^55^. The dataset contains the number of publicly announced new infections and deaths since January 16, 2020, when the first cases in Japan were discovered. To exclude the influence of new variants and vaccination, the analysis covers the data published until April 30, 2021.

#### Restaurant sales and the number of customers

The other dependent variables are restaurant sales and the number of customers. They are used as proxy variables for the business conditions of restaurants (including bars). The dataset, provided by Postas Corporation, contains daily sales and the number of customers of restaurants that have installed the cloud POS register “Postas” provided by the corporation, which covers the period from January 1, 2019 to April 30, 2021.

### Estimation methods

The estimation model in this study is a two-way fixed effects model with prefecture as the regional unit and week as the time series unit. The dataset provided by Postas Corporation is at the prefectural level. It is also the lowest level at which data on infections can be obtained due to privacy concerns. New infection cases are calculated on a weekly basis rather than a daily basis to reflect infection dynamics more accurately. For example, there is a reported lag of 6 to 12 days between onset of illness, testing, reporting to government agencies, and publication of data^8^. This lag varies depending on the number of new cases and the capacity of the public health office^56^, so there are likely to be differences in the lag among prefectures in the reporting process. This unknown length of the lag prevents us from setting the lag structure between the number of certifications issued and the change in new cases a priori. Thus, the consequent daily model would be subject to substantial measurement errors in both the independent and dependent variables. Aggregating daily data to the weekly level at least mitigates, even if it does not completely remove, the problem. In addition, some health offices report the number of infected persons collectively on a weekly basis^57^. Moreover, a variable baseline model to assess the trade-off between infection and economic activity by Fujii and Nakata use week as the unit of analysis^13^. Indeed, in line with our expectation, the weekly estimation model demonstrates lower AIC (Akaike Information Criterion) than the daily estimation model, which indicates the lower prediction errors (see Supplementary Information B.2.1). Therefore, this paper concludes to exploit the weekly time series unit.

The equations for estimating the infection prevention effect and the economic effect are as follows.

First, the infection prevention effect is estimated by the following two-way fixed-effects model. The derivation process of Eq. (1) from the SIR model is described in B.1 Derivation of the Epidemiological Equation from the SIR model of the Supplementary Information.

1 $$\begin{gathered} \ln \left( {New\;cases_{p,t} + 1} \right) = \beta_{1} \ln \left( {GZ_{p,t - 2} + 1} \right) + \beta_{2} \ln \left( {Susceptible_{p.t - 2} } \right) + \beta_{3} \ln \left( {Infectious_{p,t - 2} + 1} \right) \hfill \\ \quad \quad \quad \quad \quad \quad \quad \quad \quad + \beta_{4} \ln \left( {Customer_{p,t - 2} } \right) + \mathop \sum \limits_{i = 1}^{k} \gamma^{i} \ln \left( {Control_{p,t - 2}^{i} } \right) + c_{p} + \tau_{t} + u_{pt} \hfill \\ \end{gathered}$$

2 $$\begin{array}{*{20}c} {Susceptible_{p,t - 2} = Pop_{p} - \mathop \sum \limits_{k = 1}^{2} New\;cases_{p,t - k} } \\ \end{array}$$

3 $$\begin{array}{*{20}c} {Infectious_{p,t - 2} = \mathop \sum \limits_{k = 1}^{2} New\;cases_{p,t - k} + \mathop \sum \limits_{i = 1,i \ne p}^{47} \frac{{\mathop \sum \nolimits_{k = 1}^{2} New\;cases_{i,t - k} {*}flow_{i,p,t - k} }}{{Pop_{i} }}} \\ \end{array}$$

The subscripts $p$ and $t$ denote prefecture and week, respectively. For logged variables, we add one to the variable to avoid the issue that $log\left(0\right)$ cannot be defined. This process aims to follow the SIR model and to estimate the elasticity of effect. The outcome variable, $New \; case{s}_{p,t}$ is the published number of new infections at time $t$ in prefecture $p$. The variables on the right-hand side are the values at $t-2$. This is because it takes about six to twelve days from exposure to the virus, through the incubation period, to onset of symptoms, testing, and publication of new numbers^8,58^. This selection of the lag period for the policy effect is further supported by the AIC calculation results, which show that the estimated model based on a two-week lag has the lowest AIC of all possible models with lags of one to five weeks (see Supplementary Information B.2.2). Since the lag between exposure and announcement may vary depending on the region and time of year, analysis result is reported for a lag of one week as well as two weeks (see Supplementary Information D.2). $G{Z}_{p,t-2}$ is the cumulative number of restaurants that have received the GZ certification. The parameter ${\upbeta }_{1}$ is the treatment effect, and if this coefficient is negative and significant, it implies a high possibility of an infection-prevention effect. As described in Eq. (2), $Susceptibl{e}_{p,t-2}$ is the susceptible population that can potentially be infected, and it is defined as the population in a prefecture ($Po{p}_{p}$) minus the total number of infection cases in prefecture $p$ ($New \; case{s}_{p,t - k}$).

The variable $Infectiou{s}_{p,t-2}$ is the estimated total number of infectious people who can infect others in $p$ at $t-2$. As the derivation process is described in Eq. (3), it is the sum of the total number of infected people who existed in prefecture $p$ at $t-2$ and $t-1$ (the first term) and the total number of infected people who flowed in from other prefectures (the second term). The second term is the sum of the number of newly infected people in prefecture $i$ ($New\hspace{0.25em}case{s}_{i,t-k}$) divided by the population ($Po{p}_{i}$) multiplied by the number of people flowing into prefecture $p$ from prefecture $i$ ($flo{w}_{i,p,t-k}$) at $t-2$ and $t-1$. The identification methodology for infection dynamics across prefectures, based on a previous study by Kurahashi et al.^59^, is used to accurately measure the potential number of infected persons who can infect others at a given point in time, to more strictly control for the infection situation^59^. Since this paper assumes a trade-off in which increased economic activities come at the cost of increased infections, we also control for the number of restaurant customers, $Custome{r}_{p,t-2}$. For the control variables, we include dummy variables for the declaration of a state of emergency, school closures, and bans on large assemblies, as the previous studies indicate their effects^5–7^. We also control for the mean temperature, mean precipitation, and the number of COVID-19 test cases for infections, in accordance with the findings of review papers on meteorological impacts^60,61^. Lastly, ${c}_{p}$ and ${\uptau }_{t}$ represent prefecture and week fixed effect, respectively, and ${u}_{pt}$ is an error term.

Second, the economic effect is estimated by the following fixed-effects model.

4 $$\begin{array}{*{20}c} {Y_{pt} = {\upbeta }_{1} \ln \left( {GZ_{p,t} + 1} \right) + {\upbeta }_{2} \ln \left( {New\;cases_{p,t} + 1} \right) + \mathop \sum \limits_{i = 1}^{k} {\upgamma }^{i} \ln \left( {Control_{p,t - 2}^{i} } \right) + c_{p} + {\uptau }_{t} + u_{pt} } \\ \end{array}$$

As before, for logged variables, we add one to the variable to avoid the issue that $log\left(0\right)$ cannot be defined. For the outcome variable, ${Y}_{p,t}$, the main variable is (i) per restaurant sales and customers. For robustness and a mechanism check, (ii) the rate of increase in restaurant website visits, (iii) the rate of change in human flow by facility type, (iv) the rate of change in human flow in residential areas, and (v) the stay-home rate by age group are additionally used. $G{Z}_{p,t}$ is the cumulative number of restaurants that have received GZ certification. For the economic analysis, no lag period is exploited in estimating policy effects, because unlike data on the COVID-19 infection cases, where there is a delay between the spread of infection and the reporting of data, economic data reflects the current status of sales and the number of customers without delay. The AIC calculation supports the lag selection as the model without lag period demonstrates the lowest AIC of all possible models with lags of zero to five weeks for the main economic variable of per restaurant sales and customers (see Supplementary Information B.2.3 & B.2.4). The parameter ${\upbeta }_{1}$ is the treatment effect, and if this coefficient is positive and significant, it implies a high possibility of positive economic effects. $New case{s}_{pt}$ is the number of new infection cases in prefecture $p$ at time $t$, which we added to control for the effect of people voluntarily refraining from going out due to the spread of infection. For the control variables, we include a dummy variable for state of emergency declarations, a dummy variable for school closure, a dummy variable for bans on large assemblies, the squared term of mean temperature, mean precipitation, and the number of test cases for infections. Lastly, ${c}_{p}$ and ${\tau }_{t}$ represent prefecture and week/day fixed effect, respectively, and ${u}_{pt}$ is an error term.

### Treatment and control

To eliminate endogeneity as much as possible, the control group was selected from five neighboring prefectures with similar population density and distance from Tokyo, the epicenter of infection (see Supplementary Information E.1). This is evidenced by previous studies indicating that these geographical and urbanization factors affect the speed and duration of infection spread^62–64^. The treatment effect of infection prevention is to have the restaurants comply with the comprehensive infection-prevention guidelines through on-site checks. The estimated effect is not a comparison with the scenario when no infection prevention or economic measures were taken at all. As shown in the table (see Supplementary Information E.2), certification and subsidy policies for restaurants existed in other prefectures, but either the policies had not been launched by April 2021 or they had lower infection prevention standards and did not require on-site checks by the government.

The infection-prevention guidelines include the following five categories of measures^50^. The first is measures for customers, who can be divided into two cases: (i) entering the store, ordering, and paying, and (ii) dining and in-store use. For the rules concerning (i), disinfection equipment should be installed at the entrance of the store, partitions should be set up to separate clerks and customers at the cash register, and customers should be reminded to wear masks except when eating or drinking. For (ii) dining and in-store use, there should be space between each group and also between seats within a group. The space should be at least one meter wide, but a partition at any interval can substitute for space. Other restrictions include limiting the length of stay and serving food to individual customers rather than on platters. The second is measures for employees. It includes wearing masks at all times, checking their temperature and health condition before starting work, and regular hand disinfection and hand washing. The third is measures to ensure the hygiene of the facilities and equipment. There should be constant ventilation through ventilation equipment or open windows, and the use of hand dryers should be prohibited. The fourth is the creation and publication of a checklist to check the above infection-prevention measures and publicize the daily checks. The last is measures in the case of an infection outbreak. The guidelines stipulate that employees who are suspected of being infected should refrain from coming to work until the test results turn out to be negative, and, if necessary, information should be disclosed to prevent the spread of infection; for example, business days during which there were cases of possible infection should be disclosed. Further details are described in E.4 GZ Certification criteria of the Supplementary Information.

The treatment effect on economic activity operates through both supply and demand. On the supply side, due to the low number of infection cases, restaurant operations are maintained because the government does require restaurants to close or shorten their operation hours. In fact, except for the time of an explosive spread of the infection from December 2020 to January 2021, Yamanashi Prefecture has not declared business suspension requests, unlike other prefectures (Supplementary Information Table E.3). On the demand side, customer demand is maintained because consumers do not refrain from going out, due to the lower number of infection cases and the smaller risk of infection in certified restaurants.

## Results

**Figure 5:**
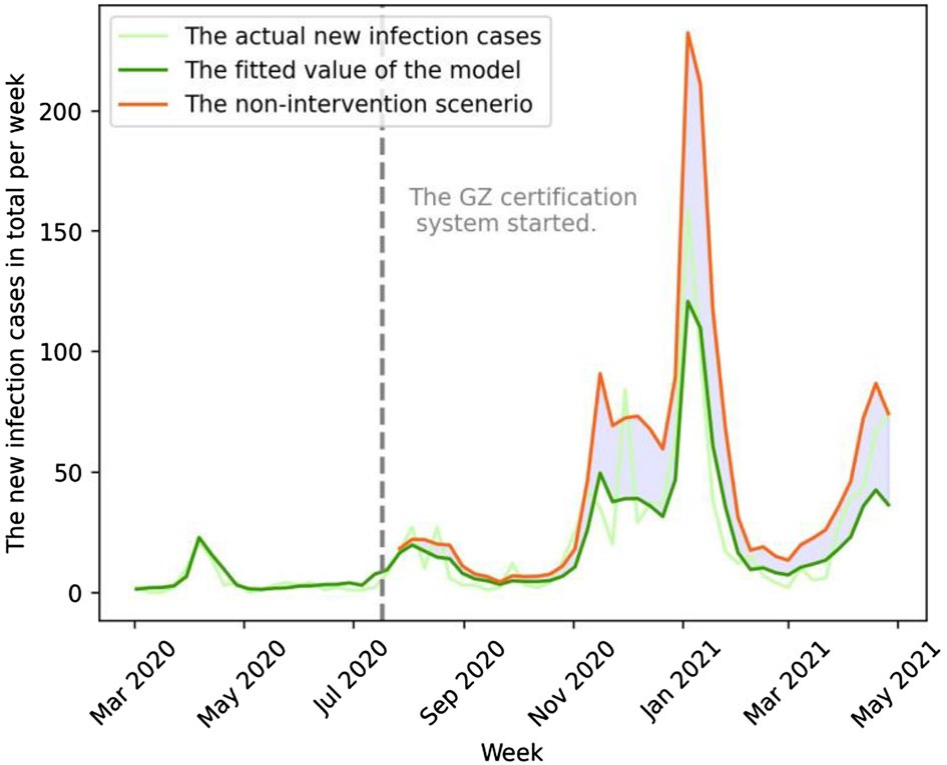
GZ-certification policy effect on the number of COVID-19 new infection cases in Yamanashi Prefecture. The vertical axis is the prefecture-wide new infection cases per week. The light-green, green, and orange lines show the actual, fitted, and non-intervention (counterfactual) scenarios, respectively. The green and orange lines are based on model (1) of Table 6 (see Supplementary Information). The green dotted line signifies the week when the GZ certification policy approved the first group of restaurants. The light-blue shaded area corresponds to the treatment effect.

**Figure 6:**
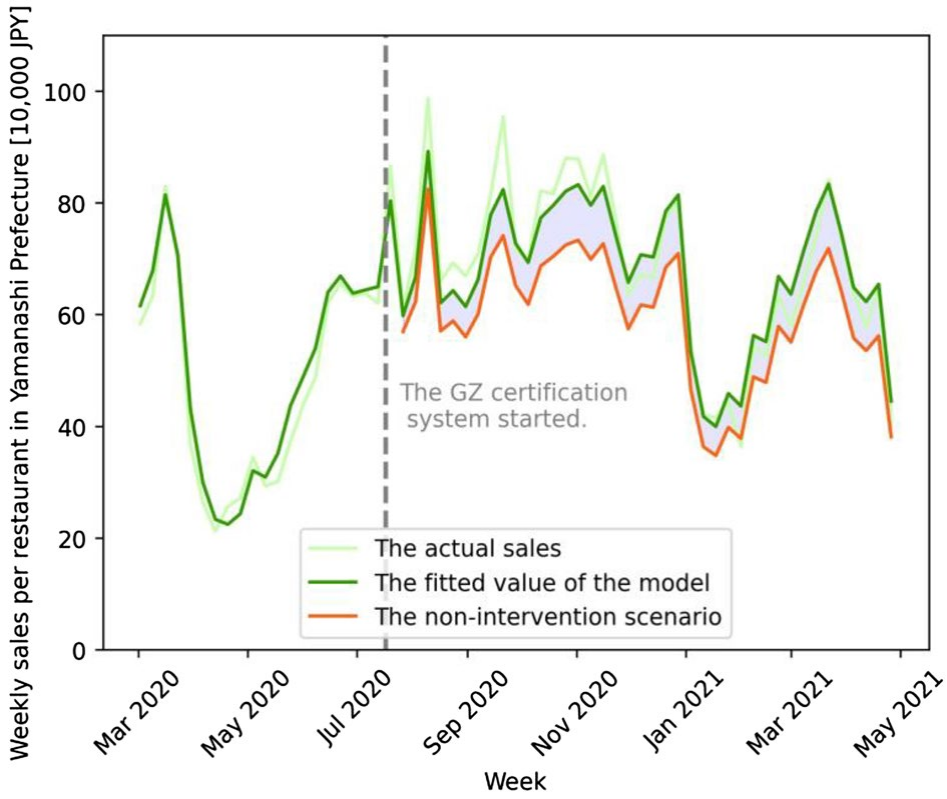
GZ-certification policy effect on restaurant sales in Yamanashi Prefecture. The vertical axis is the weekly sales per restaurant in Yamanashi Prefecture (in JPY 100,000). The light-green, green, and orange lines show the actual, fitted, and non-intervention (counterfactual) scenarios, respectively. The green and orange lines are based on model (1) of Table 8 (see Supplementary Information). The green dotted line signifies the week when the GZ certification policy approved the first group of restaurants. The light-blue shaded area corresponds to the treatment effect.

**Figure 7:**
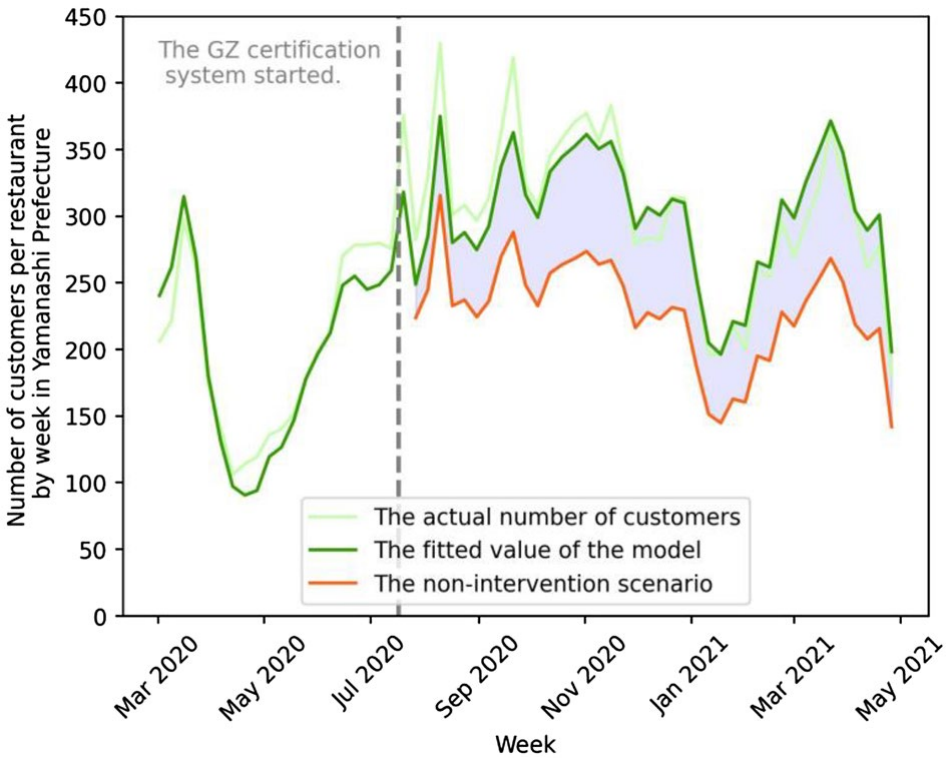
GZ-certification policy effect on the number of restaurant customers in Yamanashi Prefecture. The vertical axis is the weekly number of customers per restaurant in Yamanashi Prefecture. The light-green, green, and orange lines show the actual, fitted, and non-intervention (counterfactual) scenarios, respectively. The green and orange lines are based on model (5) of Table 8 (see Supplementary Information). The green dotted line signifies the week when the GZ certification policy approved the first group of restaurants. The light-blue shaded area corresponds to the treatment effect.

The GZ certification policy considerably decreased the number of new COVID-19 cases and increased restaurants’ sales and the number of customers in Yamanashi. From July 2020 to April 2021, the GZ certification policy reduced the number of new infection cases by approximately 45.3% (848 new cases) as compared to the non-intervention scenario (the model in column (3) of Supplementary Information D.1) (see Fig. 5). The GZ certification policy also increased total sales and the number of customers per restaurant by approximately 12.8% (3.21 million Japanese yen or $30,000, based on the 2020 average yearly exchange rate by the U.S. Internal Revenue Service) and 30.3% (2,909 customers), respectively, compared to the non-intervention scenario (the models in columns (1) and (5) of Supplementary Information Table 8) (see Figs. 6 and 7).

The treatment effects estimated above correspond to the light-blue shaded areas in the Figs. 5, 6, and 7. The areas are the sum of the difference between the fitted value and the non-intervention scenario value of the corresponding models, indicating the effect sizes in absolute values. The effect sizes on percentage bases are calculated by the sum of fitted values divided by the sum of non-intervention scenario values (both are in absolute terms).

### Statistical testing

The non-intervention scenarios are calculated through statistical analyses. For both epidemiological and economic analyses, we compare Yamanashi Prefecture, the treatment group, with its surrounding prefectures with similar external conditions for infection spread. The inclusion of two fixed effects, a time-invariant prefecture effect (e.g., population) and a prefecture-invariant time effect (e.g., infection-spread period), focuses the comparison of prefectures on the degree of infection spread, not the absolute quantity of infection cases. We use the two-tailed t-test, because the estimated OLS coefficients for the number of new infections, restaurants’ sales, and customers are known to follow normal distributions, and when the estimated coefficient is standardized by the standard error, it follows a t-distribution. Analysis methods are further discussed in the methods section and in Supplementary Information Table 5 for the summary statistics and D. Statistical Testing.

### Epidemiological effects

Our epidemiological analyses show a consistent and significant negative coefficient when we regress the log-transformed number of new infection cases with a two-week lag on the log-transformed cumulative number of GZ-certified restaurants (see Supplementary Information Table 6). The time lag is set, because previous studies have demonstrated that the time lag between the infection and the announcement of the disease is six to twelve days^8,58^. The AIC calculation results also support this lag period selection (see Supplementary Information B.2.2). For robustness, we repeated the analyses with a one-week lag and obtained similar results (see Supplementary Information Table 7). The regression results indicate that a 1% increase in the number of cumulative GZ-certified restaurants is accompanied by a 0.088% decrease in the number of new COVID-19 cases (column (1)). The estimated coefficient is statistically significant at the 1% level, with p-value 0.002. Our analyses control for the estimated number of potentially infected people, declarations of states of emergency, average rainfall (mm), average temperature (Fahrenheit), school closure, bans on large assemblies, and the number of COVID-19 tests. Since economic variables are not controlled for, the results imply that the drop in the number of infection cases was caused by the reduction in the probability of getting infected with COVID-19 in a given restaurant, even with higher economic performance (i.e., more contact opportunities) than in the neighboring prefectures. In fact, when we control for the number of restaurants’ customers (i.e., contact opportunities), the coefficient becomes 0.105%. This difference indicates that the probability of getting infected with COVID-19 in a given restaurant decreased as considerably as to offset the increase in contact opportunities in restaurants. When we repeat the analyses using the cumulative number of GZ-certified hotels along with that of certified restaurants in the same period, the coefficient attenuates but is still statistically significant, with p-value 0.008 (column (4)). We do not treat the number of certified hotels as a control variable but add it to the total number of restaurants, because the guidelines for the GZ certification in hotels mainly target dining areas within their facilities. Thus, we assume that the same infection-prevention mechanism works.

### Economic effects

Our economic analyses suggest that there are consistently significant positive coefficients when we regress the log-transformed restaurants’ sales and the log-transformed number of customers on the log-transformed cumulative number of GZ-certified restaurants (see Supplementary Information Table 8). The regression results indicate that a 1% increase in the number of cumulative GZ-certified restaurants is accompanied by 0.018% and 0.040% increases in the restaurants’ sales and number of customers, respectively (columns (1) and (5)). The estimated coefficient is statistically significant at the 1% level, with *p*-values 0.001 and 0.0001, respectively. We control for declarations of states of emergency, average rainfall (mm), average temperature (Fahrenheit), school closure, and bans on large assemblies. Given that we did not control for the number of new COVID-19 cases, we can interpret that the fewer infection cases brought by the GZ certification resulted in a smaller number of consumers refraining from going out and a smaller number of stores refraining from doing business. Even when we control for the number of infection cases, the magnitudes of the positive coefficients remain at 0.16% and 0.37%, respectively. A plausible interpretation of these results is that GZ certification may have motivated customers to visit restaurants by lowering their risk perception to possible infection. Meanwhile, the higher elasticity of the number of customers relative to that of sales indicates that per-customer spending might have been lower than before the COVID-19 crisis due to a decrease in both time spent and consumption of alcoholic beverages, which resulted from infection-prevention measures. The positive coefficient of the number of cumulative GZ-certified restaurants remains robust even if we replace restaurants’ sales and visitors with the percentage increase in restaurant website visits (see Supplementary Information Table 9).

To investigate the mechanism of the economic effects, we ran three separate regressions for (i) human mobility by facility type, (ii) human mobility by resident type, and (iii) the stay-home rate by age against the number of cumulative GZ-certified restaurants (see Supplementary Information Tables 10–14). For (i), the analysis using Google mobility data shows a statistically significant positive effect of GZ-certified restaurants on human mobility in retail and recreation (*p*-value = 0.0004) and parks (*p*-value = 0.00001), which include target facilities of the GZ-certification, and no statistically significant effects on grocery and pharmacy (*p*-value = 0.235), which are not target facilities (see Supplementary Information Table 10). In terms of (ii) human mobility by resident type, the coefficient for interprefectural mobility is positive and statistically significant (*p*-value = 0.001 (column (7))), while the positive coefficient for intercity mobility is smaller and the coefficient for intracity mobility is negative. This suggests that the GZ certification may have attracted more restaurant visitors from outside the prefecture than within the prefecture (see Supplementary Information Table 11). Finally, concerning (iii) the stay-home rate by age group, we consistently observe statistically significant negative coefficients at the 5% level for males in all generations, except in their 60 s (*p*-value = 0.086); and females aged 30 s or older, except those in their 50 s (*p*-value = 0.074) (see Supplementary Information Table 12). The coefficient for males aged 15–19 is positive, and the coefficient for females aged 15–19 and in their 20 s is negative, but not statistically significant at the 10% level. This suggests that the GZ certification policy, which lowers the psychological hurdle for consumers to go out, may have encouraged people to go out, particularly among people in their 30 s and older. In addition, we observe a statistically significant positive coefficient for the percentage of people going out at night. This suggests that the number of people going out for dinner increased due to the policy (see Supplementary Information Table 14).

## Discussion

The GZ certification policy was introduced in an effort to cut down the probability of getting infected with COVID-19 at given facilities, primarily restaurants, while maintaining economic activities. The results of our analyses are in line with the policy objectives: The GZ certification policy likely dropped the number of infection cases while increasing restaurants’ sales and the number of customers.

The reason why Yamanashi Prefecture was able to reduce the infection probability substantially, relative to neighboring prefectures, is that the GZ-certification increased the percentage of restaurants that complied with infection-prevention guidelines, compared to the cases where prevention was entrusted to each restaurant. As for mechanisms, we assume that multiple factors incentivized restaurants to apply for and keep abiding by the certification guidelines. For example, subsidies lowered the investment cost of equipment necessary for certification. The guidelines stipulate comprehensive infection-control measures, including those reported to be effective by previous studies, such as wearing masks^34,35^, securing space between seats^32^, limiting maximum capacity^15,37^, and installing functional partitions and ventilation facilities^11,29–31^, all of which incur additional costs for owners to accomplish^50^. By covering the cost for implementing such measures, the subsidy scheme may have facilitated restaurants’ applications for the certification.

Also, the certification reduced information asymmetry about infection prevention between restaurants and customers. Since the degree to which a restaurant practices infection control is not completely clear to customers prior to their visit, they often judge via brand trust^44^. The certification by the government provided consumers with a clearer decision-making tool, which led consumers to demand certified establishments, leading more restaurants to apply for the certification. This consumer preference for restaurants with infection-prevention measures is consistent with the results of several recent studies^41–47^. In addition, the third-party on-site inspections have lowered restaurants’ incentives to deviate from compliance with the guidelines. Without the on-site verification, certified restaurants may have had an incentive to deviate from guideline compliance, because following the guidelines, such as reducing the number of seats, would undermine their profits. Such a condition would have substantially diminished the effectiveness of infection-prevention measures. In fact, the majority of prefectures during the study period only required self-reporting documents for accreditation, and the guidelines were not fully operational^52^ (see Supplementary Information E.2).

Regarding the positive economic spillovers, we assume the following two mechanisms resulting from a third-party certification policy. First, the small number of infection cases supported the prefecture’s decision to maintain restaurants’ usual operating hours, allowing them to make sales, while restaurants without proper measures faced shortened operating hours. This is evidenced by the fact that Yamanashi Prefecture had fewer business closure requests than their neighboring prefectures (see Supplementary Information E.3). Second, the policy made consumers feel more at ease when dining out. This overlaps with the reason why the effectiveness of infection prevention was confirmed: Consumers perceived the value of service at restaurants that thoroughly implemented hygiene measures, such as partitions and distance between seats and mask-wearing by employees^41–47^. Consequently, consumers continued to visit restaurants nearly as much as they did pre-pandemic, which helped businesses to survive.

This research has two main contributions. First, we have exploited a natural experiment research design to credibly evaluate the epidemic and economic effects of a third-party certification policy. As described in the introduction section, many econometric analyses have simulated the epidemiological effects of non-pharmaceutical interventions in restaurants. Nonetheless, most of them have relied on simulation studies^11,12,29,30^, and only a few have examined the policy effect by exploiting a natural experiment^34–36^. Even when natural experiments are used, the results are likely to be biased, because researchers often do not control for factors that can significantly affect the way infection spreads, such as population density, distance from urban areas, and the number of infected people that would have existed in an area at a given time, all of which are demonstrated to have a considerable impact on infection spread^62–64^. We have overcome these potential endogeneity issues by incorporating the following five approaches. First, we constructed an estimation model based on the SIR model, a classical mathematical model of epidemiology^54^, to correctly capture the infection dynamics. Second, we included prefecture and time fixed effects to control for heterogeneity across prefectures and time periods. Third, we limited our sample to prefectures with similar population density and distance from metropolitan areas, the epicenters of infection, to construct the control group (See Supplementary Information E.1). Fourth, we take into account the potential migration of infected people between prefectures by deploying the method developed by Kurahashi et al.^59^. Their model enables us to fix the number of potentially infected people, and therefore we can examine the extent to which infection spreads. Lastly, we control for other policies that could influence the outcome variables as much as possible. For example, we introduce school closure dummy variables and bans on large assembly dummy variables. We also confirm the similarity in the policies of the treatment and control prefectures except for the presence of the GZ certification by qualitatively studying restaurant-related policies.

The second contribution is that we have provided the first evidence, to the best of our knowledge, of the effectiveness of a third-party certification policy for restaurants in mitigating the trade-off between infection prevention and economic sustainability. We believe this finding plays an important role in the fight against the epidemic, as policymakers are increasingly required to seek a balance between infection risks and economic sustainability in the long run, given the uncertain prospects of the pandemic and finite administrative budgets. To this end, some countries, such as Japan and Singapore^48^, have implemented third-party certification policies, but little is known about their effectiveness. We have indicated that effective infection-mitigating measures can be developed not necessarily through business-regulatory policies that impose obligations and financial burdens on restaurants, but through information policies that take advantage of consumers’ demand by publicizing certification status, and through subsidy policies that help owners to install necessary equipment, which is in line with a recent study^37^. Moreover, we have found that restaurants have survived the pandemic era by operating in-store without resorting to takeout or drone delivery, which was the main focus of the previous study^38–40^.

A limitation of this study is that the route of transmission is not fully known. This is because the study examines the effect of the policy using the geographical unit of prefectures, rather than, for example, individual data on the use of specific restaurants and whether individuals are infected or not. Our study controlled as much as possible for differences between treatment and control prefectures in terms of routes and policies that could affect infection status, but there may be differences in infection routes other than through restaurants. Future studies are expected to identify the policy impacts on the route of infection by deploying different datasets. For policymakers to review the policy impacts, they should also refer to previous studies, especially those cited in this paper, that simulate droplet infection in indoor spaces and elucidate the effectiveness of related measures (masks, ventilation, school closures, etc.).

## Conclusion

The trade-off between infection control and business maintenance in restaurants has been a major concern for policymakers all over the world. This paper demonstrates the impact of a third-party certification system that requires restaurants to comply with infection-control guidelines on the number of infected people and restaurant sales in the area where the system is introduced, using a case study in Yamanashi Prefecture, Japan. The research design is based on a difference-in-differences method, taking advantage of the fact that the number of certified stores gradually increased in Yamanashi Prefecture, whereas the certification system had not yet substantially diffused in the five neighboring prefectures. We have constructed an estimation model to remove endogeneity in the infection spread apart from policy effects, such as population density, distance to Tokyo, and movement of potentially infected persons between prefectures.

Our study provides evidence that a third-party certification policy on infectious disease control in restaurants has mitigated the trade-off between infection prevention and economic operation. The policy successfully reduced the number of infections in the targeted prefecture by roughly 45.3%, while increasing total sales and the number of customers per restaurant by roughly 12.8% and 30.3%, respectively. We reason that the mechanisms for the epidemiological effect are that (1) the subsidies for installing necessary facilities and equipment have eliminated the financial hurdle for owners; (2) the certification has successfully incorporated infection-control status into the market principle for restaurants, which provided an incentive for restaurants entrepreneurs to acquire certification; and (3) on-site administrative checks have maintained the intensity of infection-control measures. Economic effects are deemed to be attributed to the fact that (1) the certification of restaurants has catered to consumers’ demand for sanitary practices, and (2) the epidemiological effect to keep the infection cases low has realized the fewer administrative business suspensions.

We have contributed to the research field in two ways. First, we exploited a natural experiment to credibly estimate the epidemiological and economic effects of a certification policy that requires compliance with infection-control guidelines in restaurants. Not only the research design of difference-in-differences but also the SIR-model-based estimation accompanied by rigorous endogeneity control have ensured the robustness of the findings. Second, we have presented the first evidence amid the fight against the COVID-19 pandemic that a certification policy can beat the trade-off between infection control and business operation.

This study implies four practical and managerial implications for policymakers and restaurant entrepreneurs. First, unlike business-restricting measures, such as lockdowns and shortening hours, the certification policy pinpoints and substantially reduces the infection risks of short- and long-range airborne transmission, thereby diminishing infection cases while maintaining business operations. Second, the key for the effect resides not in the establishment of the guidelines themselves, but rather in the on-site administrative verification to uphold compliance. Third, the certification policy is a versatile tool without high administrative costs, and therefore it can be applied to many regions. Last, restaurants do not necessarily need to operate a take-out business to maintain a certain level of sales by implementing strategies to improve infection-control measures inside the restaurant establishment. In concluding the study, we hope that policymakers will make good use of the findings from this research in balancing infection prevention and economic sustainability in the current as well as future pandemics.

## Supplementary Information

Supplementary Information.

## Data Availability

The datasets generated and analyzed are available in the Github repository, https://github.com/jhirota/GZ. Restrictions apply, however, to the availability of the following raw datasets, which were used under license for the study, and so are not publicly available: (i) the list of restaurant names, addresses, and GZ certification date provided by Yamanashi Prefecture; (ii) the number of restaurants used when constructing sales and the number of customers panel data provided by Postas Corporation; and (iii) the population moving from one prefecture to another (for 47 prefectures) per day provided by Agoop Corporation.
