# Supplementary material for: Investigating the epidemiological and economic effects of a third-party certification policy for restaurants with COVID-19 prevention measures
Source: Sci Rep. 2023 May 11;13:7655. doi: 10.1038/s41598-023-34498-w (PMC10173234; doi:10.1038/s41598-023-34498-w)
Supplement: Supplementary file 1 — Supplementary Information. [file 41598_2023_34498_MOESM1_ESM.pdf]

Supplementary Information

Investigating the epidemiological and economic effects of a third-party certification for restaurants with COVID-19 prevention measures

Kazuya Hirokawa, Jumpei Hirota, Daiji Kawaguchi, Yusuke Masaki, Chiaki Onita

In this online appendix, we summarize the sub-evidence for the main article. First, we describe the data. Second, we derive the estimation equation. Third, we list the basic statistics for each variable. Fourth, we present our regression results. Finally, we provide supplementary information about the treatment effects.

Table of Contents

A. Data

1. COVID-19 policy dummy variables
2. COVID-19 test cases
3. Weather data
4. Human mobility inflow and outflow by prefecture
5. Restaurant information views online
6. Human mobility by facility type
7. Human mobility across regions
8. Stay-home rate

B. Estimation Equation

1. Derivation of the Epidemiological Equation from the SIR model
2. Model selection of the time lag for the policy effects (AIC)

C. Basic Statistics

1. Summary statistics of analysis
2. Cumulative number of the GZ-certified restaurants

D. Statistical Testing

1. Infection prevention effects (2 week lag analysis)
2. Infection prevention effects (1 week lag analysis)
3. Economic effects (restaurants' sales and the number of customers)
4. Restaurant information views online
5. Human mobility by facility type

6. Inter-regional Mobility

7. Stay-home rate

E. Treatment Effect

1. Comparison of Treatment and Control prefecture
2. Comparison of policies in Treatment and Control prefecture
3. List of Business suspension request
4. Yamanashi Green Zone Certification criteria

A. Data

Our data used for control variables (A.1–A.4) in the main analyses (D.1, D.3) and dependent variables (A.5–A.8) in the robustness check (D.4–D.7) come from multiple sources.

A.1 COVID-19 policy dummy variables

For the emergency declaration dummy variable, we use data on the progress of the government's response as summarized by Tottori Prefecture on its website for new COVID-19 infections. The site lists the prefectures that are subject to the issuance, change, and cancellation of emergency declarations in chronological order. Based on this information, we determined whether each prefecture was under a state of emergency declaration at a certain time. For the school closure dummy variable, we use data on information of school closures for the national government and each prefecture in the time-series news archives on NHK's special website for COVID-19. The variable was set to be 1 only when schools are closed in the entire prefecture. For municipality or school-level closure, the variable is set to be 0. The gathering restriction dummy variable is created based on the governor's press conferences and updates on COVID-19 in each prefecture. The variable takes a value of one if there is a restriction on event capacity of 5,000 people or less and a capacity ratio of 50% or less in each prefecture, and takes a value of zero if either of these criteria is not met. In cases where the prefectural criteria are based on the guidelines of the respective industry, the dummies are created based on the common guidelines of event-related industries.

A.2 COVID-19 test cases

In the analysis of the infection prevention effect, we use the data on the number of COVID-19 test cases published by each prefecture. As of 2020, the number of tests in each prefecture varies greatly in Japan, particularly because of varying capacities for conducting tests across prefectures. If the number of tests itself is small, the number of new infection cases may be underestimated.

A.3 Weather data

For the weather data of temperature and precipitation, we use the daily weather observation data of observatories in each prefecture using the "Past Weather Data Search" of the Japan Meteorological Agency. The average rainfall and the average temperature are used as the representative values. When extracting the data from the database, several municipalities with observatories are chosen from several municipalities with the top population. In detail, Yamanashi Prefecture is represented by Kofu and Kawaguchiko; Nagano Prefecture by Nagano, Matsumoto, Ueda, and Iida; Shizuoka Prefecture by Hamamatsu, Shizuoka, and Fuji; Gunma Prefecture by Maebashi and Isesaki; Ibaraki Prefecture by

Tsukuba, Mito, and Hitachi; and Tochigi Prefecture by Utsunomiya and Oyama. The weather data for each prefecture is the average of the data for the municipalities belonging to each prefecture.

A.4 Human mobility inflow and outflow by prefecture

We use Agoop's paid data for the human flow within each prefecture and the human flow from outside the prefecture into each prefecture. The data is drawn from users' GPS information held by Agoop. It is the data that calculates the entire human flow from a sample of the number of people who existed at a certain coordinate at a certain time. The data provides information on not only the total number of people in a prefecture, but also the movement of people from a specific prefecture to a specific prefecture/municipality. This data is used to estimate the number of potentially infectious mobility coming from other prefectures as shown in the susceptible-infectious-recovered (SIR) model, rather than as an objective variable.

A.5 Restaurant information views online

We also use data on the rate of increase in the number of restaurant information views online per week, which is available on V-RESAS published by the Cabinet Office of Japan. The rate of increase or decrease in the restaurant information views online compared to the same week in 2019 is disclosed for each prefecture. The original data is held by Retty Inc., which operates Japan's largest word-of-mouth gourmet service. This data is used as the dependent variable in the robustness check (D.4 Restaurant information views online)

A.6 Human mobility by facility type

For the mobility data, we also use the "COVID-19: Community Mobility Report" published by Google. The data reveals the rate of increase or decrease in human flow in six types of locations ("retail and recreation," "grocery and pharmacy," "parks," "transit stations," "workplaces," and "residential") by country and region/prefecture. The median value for each day of the week for the five-week period from January 3 to February 6, 2020 is used as the baseline for the rate of change. Thus, the daily data is the rate of change from the base values for each day of the week.This data is used as the dependent variable in the robustness check (D.5 Human mobility by facility type)

A.7 Human mobility across regions

We also used human flow data on the rate of increase or decrease in the mobility by region (intracity, intercity, and interprefectural), compared to the same week in 2019 for each prefecture. This data is available on V-RESAS. This data is used as the dependent variable in the robustness check (D.6 Inter-regional Mobility) to examine the impact on human flow in and across the prefectures.

A.8 Stay-home rate

We use the data on stay-home rate, which the Mizuno Laboratory of the National Institute of Informatics and the Graduate University for Advanced Studies publish. The data is collected by age group and time, based on the population data estimated in real-time from the information of about 78 million base stations of DOCOMO, a major Japanese telecommunication company. They define the number of

people going out from residential areas as

$$The\ number\ of\ people\ going\ out = daytime\ population - nighttime\ population$$

and

$$The\ Stay-home\ rate = 1 - \frac{Number\ of\ people\ who\ go\ out\ from\ 9:00\ to\ 18:00\ on\ a\ given\ day * average\ time\ spent\ out}{Number\ of\ people\ who\ go\ out\ from\ 9:00\ to\ 18:00\ on\ a\ normal\ day * average\ time\ spent\ out}$$

This data is used in the robustness check (D.8 Stay-home rate).

B Estimation equation

B.1 Derivation of the Epidemiological Equation from the SIR model

The epidemiological analyses are based on the SIR-applied fixed effect model. This section shows the process of how the equation ((1) in Methods in the main article) is derived from the SIR model.

The SIR model captures the epidemic dynamics of infectious diseases that spread directly from person to person. The model estimates the infection status in three stages: Susceptible, Infectious, and Removed. Susceptible refers to the state of being susceptible to infection, Infectious refers to the state of being infected, and Removed refers to the state of having recovered or gained immunity or having died.

$$\frac{dI(t)}{dt} = \beta S(t)I(t) - \gamma I(t)$$

β denotes infectivity, and γ denotes recovery or isolation rate. In other words, the rate of change of the Infectious population is dependent on the interactions between the Susceptible and Infectious populations, the infectivity of the virus, and the rate at which the Infectious move to the Removed population. We can obtain an expression for the number of new COVID-19 cases in a given time period by rewriting the ordinary differential equation as:

$$COVID = \beta SI$$

Taking logarithms on both sides, we get:

$$ln(COVID) = ln\beta + lnS + lnI$$

A previous study has shown that the model that assumes that the proportion of susceptible people who can be infected and the proportion of infectious people who can infect others are not equal to one, but vary depending on the situation, can more accurately estimate the number of newly infected people (Law, K.B., Peariasamy, K.M., Gill, B.S. et al 2020). Therefore, we transform the model by adding coefficients to the Susceptible and Infectious variables as follows

$$ln(COVID) = ln\beta + \delta_2 lnS + \delta_3 lnI$$

In order to adapt this basic estimation equation to changes in external circumstances that affect the infection cases, we add control variables such as economic activity variables and weather conditions. In

addition, the purpose of the GZ certification policy that we want to estimate is to reduce the infectivity (β) that can be transferred from one infected person to another. Therefore, we redefine the infectivity (β) as follows to derive the main estimation model.

$$ln\beta = \alpha_1 lnGZ + u$$

$$ln(COVID) = \alpha_1 lnGZ + \delta_2 lnS + \delta_3 lnI + \delta_4 lnControl + u$$

B.2 Model selection of the time lag for the policy effects (AIC)

B.2.1 Model Selection (AIC) for Infection prevention effects: Unit of time series (Weekly and Daily)

Table 1: Model Selection of the time lag (Weekly and Daily): The COVID-19 new infection cases and the Green Zone certification

	Dependent variable:	
	New infection cases, log (Weekly)	New infection cases, log (Daily)
	(1)	(2)
Cumulative GZ-certified restaurants, log (2weeks/14days lag)	-0.126^{***}	-0.147^{***}
	(0.028)	(0.035)
Unit of time series	Weekly	Daily
AIC	750.29	5561.76
Observations	396	2,729
Adjusted R^2	0.914	0.812

Notes: *p<0.1; **p<0.05; ***p<0.01 The dependent variable is the log-transformed value of the number of new infection cases. The unit of analysis is prefecture and week for the first column (1) and day for the second column (2). The fixed effects are introduced in all models. For the observations, six prefectures are targeted. The values in parentheses are cluster-robust standard errors. Clustering is at the prefecture level. The following variables are included as covariates: Infectious(log), Susceptible(log), State of Emergency dummy, Tests(log), Customers per restaurant(log), Average temperature(log), Average rainfall(log), School closure dummy, Gathering restriction dummy. Cumulative GZ-certified restaurants, log is the log-transformed value of the number of cumulative certified-GZ restaurants. Infectious(log) is the log-transformed value of the number of potentially infected people. Susceptible(log) is the log-transformed value of the total number of susceptible population. State of Emergency is the dummy variable that takes the value 1 if the state of emergency is declared. Tests(log) is the log-transformed value of the number of COVID-19 tests. Customers per restaurant(log) is the log-transformed value of the number of customers per restaurant. Average temperature(log) is the log-transformed value of the mean temperature (Fahrenheit degrees). Average rainfall(log) is the log-transformed value of the aggregated rainfall (in millimeters). School closure is the dummy variable that takes the value 1 if the school closure is declared. Gathering restriction is the dummy variable that takes the value 1 if the large-scale gathering restriction is declared. For the variables that take absolute value 0 (New infection cases(log), Cumulative GZ-certified restaurants, log, Infectious(log), and Tests(log)), we add value 1 before log-transforming to avoid the logarithm of 0.

B.2.2 Model Selection (AIC) for Infection prevention effects: The COVID-19 new infection cases

Table 2: Model Selection of the time lag (Weekly data) : The COVID-19 new infection cases and the Green Zone certification

	Dependent variable:								
	New infection cases, log								
	(1)	(2)	(3)	(4)	(5)	(6)	(7)	(8)	(9)
Cumulative GZ-certified restaurants, log (2weeks lag)	−0.126***					−0.070	−0.185	−0.066	0.096
	(0.028)					(0.126)	(0.161)	(0.209)	(0.182)
Cumulative GZ-certified restaurants, log (1week lag)		−0.127***				−0.057	−0.041	−0.185	−0.061
		(0.029)				(0.145)	(0.147)	(0.202)	(0.229)
Cumulative GZ-certified restaurants, log (3weeks lag)			−0.123***				0.100	0.431**	0.134
			(0.028)				(0.084)	(0.154)	(0.213)
Cumulative GZ-certified restaurants, log (4weeks lag)				−0.123***				−0.309	−0.910***
				(0.028)				(0.153)	(0.090)
Cumulative GZ-certified restaurants, log (5weeks lag)					−0.113**				0.620**
					(0.028)				(0.157)
Unit of time series					Weekly				
AIC	750.29	750.31	751.05	750.52	753.26	752.23	754.05	754.77	751.76
Observations	396	396	396	396	396	396	396	396	396
Adjusted R^2	0.914	0.914	0.913	0.914	0.913	0.913	0.913	0.913	0.914

Notes: *p<0.1; **p<0.05; ***p<0.01 The dependent variable is the log-transformed value of the number of new infection cases. The unit of analysis is prefecture and week, and the fixed effects are introduced in all models. For the observations, six prefectures are targeted. The values in parentheses are cluster-robust standard errors. Clustering is at the prefecture level. The following variables are included as covariates: Infectious(log), Susceptible(log), State of Emergency dummy, Tests(log), Customers per restaurant(log), Average temperature(log), Average rainfall(log), School closure dummy, Gathering restriction dummy. Cumulative GZ-certified restaurants, log is the log-transformed value of the number of cumulative certified-GZ restaurants. Infectious(log) is the log-transformed value of the number of potentially infected people. Susceptible(log) is the log-transformed value of the total number of susceptible population. State of Emergency is the dummy variable that takes the value 1 if the state of emergency is declared. Tests(log) is the log-transformed value of the number of COVID-19 tests. Customers per restaurant(log) is the log-transformed value of the number of customers per restaurant. Average temperature(log) is the log-transformed value of the mean temperature (Fahrenheit degrees). Average rainfall(log) is the log-transformed value of the aggregated rainfall (in millimeters). School closure is the dummy variable that takes the value 1 if the school closure is declared. Gathering restriction is the dummy variable that takes the value 1 if the large-scale gathering restriction is declared. For the variables that take absolute value 0 (New infection cases(log), Cumulative GZ-certified restaurants, log, Infectious(log), and Tests(log)), we add value 1 before log-transforming to avoid the logarithm of 0.

B.2.3 Model Selection (AIC) for Economic effects: Restaurants' sales (POS)

Table 3: Model Selection of the time lag: Restaurants' sales (POS) and the Green Zone certification

	Dependent variable:					
	Sales per restaurant, log					
	(1)	(2)	(3)	(4)	(5)	(6)
Cumulative GZ-certified restaurants, log (0day lag)	0.016***					
	(0.003)					
Cumulative GZ-certified restaurants, log (7days lag)		0.016***				
		(0.003)				
Cumulative GZ-certified restaurants, log (14days lag)			0.015***			
			(0.003)			
Cumulative GZ-certified restaurants, log (21days lag)				0.014***		
				(0.003)		
Cumulative GZ-certified restaurants, log (28days lag)					0.014***	
					(0.003)	
Cumulative GZ-certified restaurants, log (35days lag)						0.013**
						(0.003)
AIC	-4916.94	-4912.92	-4905.61	-4899.11	-4892.43	-4883.96
Observations	5,106	5,106	5,106	5,106	5,106	5,106
Adjusted R^2	0.922	0.922	0.922	0.922	0.922	0.922

Notes: *p<0.1; **p<0.05; ***p<0.01 The dependent variable is the log-transformed value of POS sales per restaurant. The unit of analysis is prefecture and day, and the fixed effects are introduced in all models. For the observations, six prefectures are targeted. The values in parentheses are cluster-robust standard errors. Clustering is at the prefecture level. The following variables are included as covariates: State of Emergency dummy, The number of new COVID-19 cases(log), Average temperature(log), Average rainfall(log), School closure dummy, Gathering restriction dummy. Cumulative GZ-certified restaurants(log) is the log-transformed value of the number of cumulative certified-GZ restaurants. State of Emergency is the dummy variable that takes the value 1 if the state of emergency is declared. The number of new COVID-19 cases(log) is the log-transformed value of the daily number of infection cases. Average temperature(log) is the log-transformed value of the mean temperature (Fahrenheit degrees). Average rainfall(log) is the log-transformed value of the aggregated rainfall (in millimeters). School closure is the dummy variable that takes the value 1 if the school closure is declared. Gathering restriction is the dummy variable that takes the value 1 if the large-scale gathering restriction is declared. For the variables that take absolute value 0 (Cumulative GZ-certified restaurants(log), and The number of new COVID-19 cases(log)), we add value 1 before log-transforming to avoid the logarithm of 0.

B.2.4 Model Selection (AIC) for Economic effects: the number of Restaurants' customers (POS)

Table 4: Model Selection of the time lag: The number of Restaurants' customers (POS) and the Green Zone certification

	Dependent variable:					
	Customers per restaurant, log					
	(1)	(2)	(3)	(4)	(5)	(6)
Cumulative GZ-certified restaurants, log (0day lag)	0.037^{***}					
	(0.005)					
Cumulative GZ-certified restaurants, log (7days lag)		0.037^{***}				
		(0.005)				
Cumulative GZ-certified restaurants, log (14days lag)			0.036^{***}			
			(0.005)			
Cumulative GZ-certified restaurants, log (21days lag)				0.035^{***}		
				(0.005)		
Cumulative GZ-certified restaurants, log (28days lag)					0.034^{***}	
					(0.005)	
Cumulative GZ-certified restaurants, log (35days lag)						0.033^{***}
						(0.005)
AIC	-5887.78	-5862.07	-5826.23	-5795.83	-5766.27	-5734.13
Observations	5,106	5,106	5,106	5,106	5,106	5,106
Adjusted R^2	0.937	0.936	0.936	0.935	0.935	0.935

Notes: *p<0.1; **p<0.05; ***p<0.01 The dependent variable is the log-transformed value of the number of customers per restaurant. The unit of analysis is prefecture and day, and the fixed effects are introduced in all models. For the observations, six prefectures are targeted. The values in parentheses are cluster-robust standard errors. Clustering is at the prefecture level. The following variables are included as covariates: State of Emergency dummy, The number of new COVID-19 cases(log), Average temperature(log), Average rainfall(log), School closure dummy, Gathering restriction dummy. Cumulative GZ-certified restaurants(log) is the log-transformed value of the number of cumulative certified-GZ restaurants. State of Emergency is the dummy variable that takes the value 1 if the state of emergency is declared. The number of new COVID-19 cases(log) is the log-transformed value of the daily number of infection cases. Average temperature(log) is the log-transformed value of the mean temperature (Fahrenheit degrees). Average rainfall(log) is the log-transformed value of the aggregated rainfall (in millimeters). School closure is the dummy variable that takes the value 1 if the school closure is declared. Gathering restriction is the dummy variable that takes the value 1 if the large-scale gathering restriction is declared. For the variables that take absolute value 0 (Cumulative GZ-certified restaurants(log), and The number of new COVID-19 cases(log)), we add value 1 before log-transforming to avoid the logarithm of 0.

C Basic Statistics

C.1 Summary statistics of analysis

Table 5: Summary Statistics

Statistic	N	Mean	St. Dev.
New cases per day	408	76.061	122.493
Number of customers per restaurant	408	254.371	86.767
Sales per restaurant	408	585,798.500	174,182.800
Average temperature	408	56.563	14.107
Average rainfall	408	3.926	5.531
Infectious	408	172.859	260.401
Susceptible	408	2,205,422.000	877,185.900
Number of COVID-19 tests	408	1,711.466	2,655.616

C.2 Cumulative number of the GZ-certified restaurants

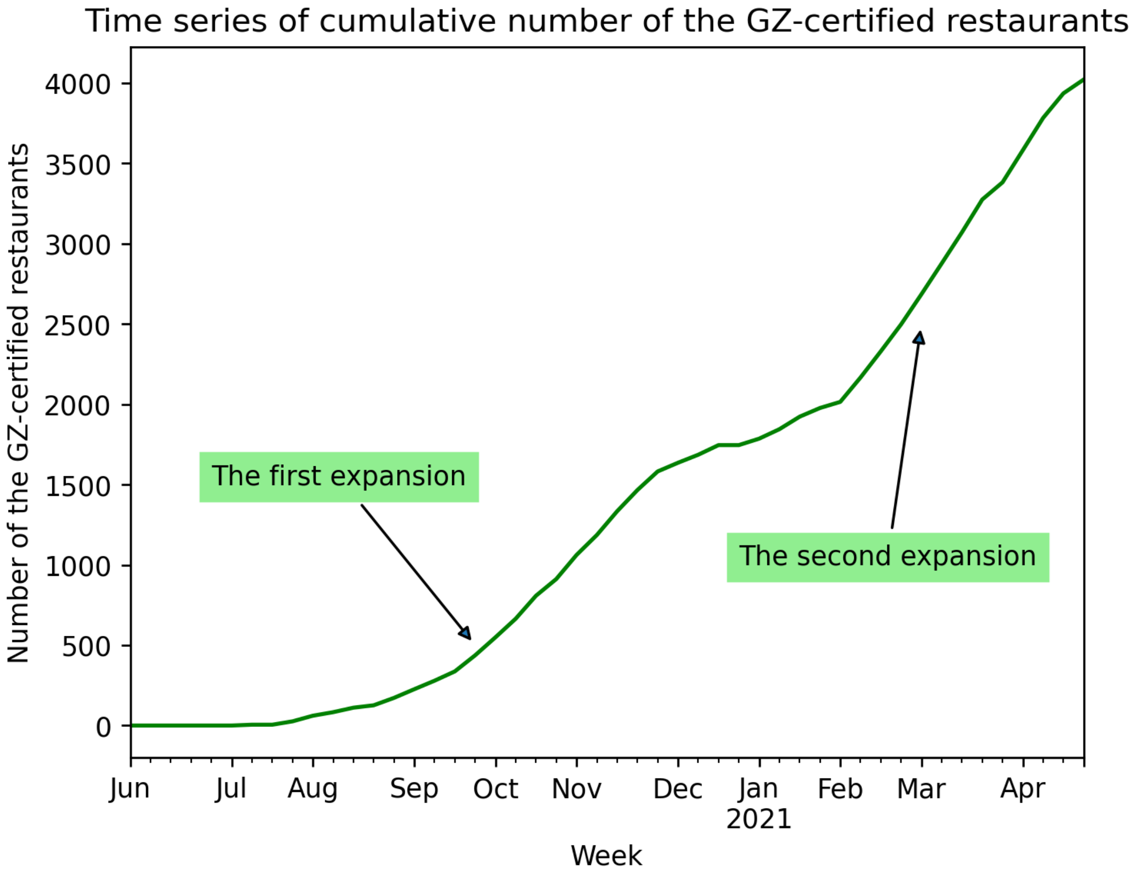

D Statistical Testing

D.1 Infection prevention effects (2 week lag analysis)

Table 6: The COVID-19 new infection cases (2 week lag) and the Green Zone certification

	Dependent variable:				
	New infection cases (2 week lag), log				
	(1)	(2)	(3)	(4)	(5)
Cumulative GZ-certified restaurants, log	−0.088***	−0.105***	−0.107***		−0.126***
	(0.015)	(0.024)	(0.025)		(0.028)
Cumulative GZ-certified restaurants and hotels, log				−0.104***	
				(0.024)	
Infectious, log	0.558***	0.569***	0.566***	0.565***	0.544***
	(0.048)	(0.045)	(0.040)	(0.040)	(0.042)
Susceptible, log	−45.353	−45.843	−44.997	−61.487	32.518
	(69.254)	(92.506)	(89.342)	(82.870)	(121.902)
State of Emergency	0.030	0.141	0.085	0.096	−0.183
	(0.223)	(0.198)	(0.180)	(0.180)	(0.200)
Tests (2 week lag), log	0.047*	0.051*	0.049**	0.050**	0.052**
	(0.021)	(0.021)	(0.019)	(0.019)	(0.018)
Customers per restaurant, log		0.550	0.548	0.612	0.540
		(0.419)	(0.449)	(0.446)	(0.480)
Average temperature, log			−0.342	−0.344	−0.317
			(0.470)	(0.466)	(0.501)
Average rainfall, log			−0.107	−0.104	−0.115
			(0.058)	(0.058)	(0.061)
School closure					−0.252
					(0.275)
Gathering restriction					0.336**
					(0.083)
Prefecture FE	X	X	X	X	X
Week FE	X	X	X	X	X
Observations	396	396	396	396	396
R^2	0.929	0.930	0.930	0.930	0.931
Adjusted R^2	0.913	0.913	0.913	0.913	0.914

Notes: *p<0.1; **p<0.05; ***p<0.01 The dependent variable is the log-transformed value of the number of new infection cases (2 week lag). The unit of analysis is prefecture and week, and the fixed effects are introduced in all models. For the observations, six prefectures are targeted, and the period of analysis is for 66 weeks from the third week of January, 2020 to the third week of April, 2021. The values in parentheses are cluster-robust standard errors. Clustering is at the prefecture level. Cumulative GZ-certified restaurants, log is the log-transformed value of the number of cumulative certified-GZ restaurants. Cumulative GZ-certified restaurants and hotels, log is the log-transformed value of the number of cumulative certified-GZ restaurants and hotels. Infectious, log is the log-transformed value of the number of potentially infected people. Susceptible, log is the log-transformed value of the total number of susceptible population. State of Emergency is the dummy variable that takes the value 1 if the state of emergency is declared. Tests (2 week lag), log is the log-transformed value of the number of COVID-19 tests. Customers per restaurant, log is the log-transformed value of the number of customers per restaurant. Average temperature, log is the log-transformed value of the mean temperature (Fahrenheit degrees). Average rainfall, log is the log-transformed value of the aggregated rainfall (in millimeters). School closure is the dummy variable that takes the value 1 if the school closure is declared. Gathering restriction is the dummy variable that takes the value 1 if the large-scale gathering restriction is declared. For the variables that take absolute value 0 (New infection cases (2 week lag), log, Cumulative GZ-certified restaurants, log, Cumulative GZ-certified restaurants and hotels, log, Infectious, log, and Tests (2 week lag), log), we add value 1 before log-transforming to avoid the logarithm of 0.

D.2 Infection prevention effects (1 week lag analysis)

Table 7: COVID-19 new cases (1 week lag) and the Green Zone certification

	Dependent variable:				
	New infection cases (1 week lag), log				
	(1)	(2)	(3)	(4)	(5)
Cumulative GZ-certified restaurants, log	−0.083***	−0.094***	−0.093***		−0.108***
	(0.015)	(0.020)	(0.019)		(0.022)
Cumulative GZ-certified restaurants and hotels, log				−0.090***	
				(0.018)	
Infectious, log	0.562***	0.575***	0.574***	0.573***	0.560***
	(0.048)	(0.043)	(0.041)	(0.041)	(0.041)
Susceptible, log	−107.523	−102.924	−99.505	−113.259	−29.163
	(75.138)	(90.837)	(86.466)	(80.316)	(132.249)
State of Emergency	0.009	0.089	0.090	0.100	−0.123
	(0.185)	(0.194)	(0.213)	(0.215)	(0.191)
Tests (1 week lag), log	0.049*	0.053**	0.053*	0.054*	0.052*
	(0.021)	(0.020)	(0.022)	(0.021)	(0.022)
Customers per restaurant, log		0.395	0.365	0.422	0.333
		(0.412)	(0.412)	(0.417)	(0.413)
Average temperature, log			0.355	0.352	0.397
			(0.433)	(0.445)	(0.413)
Average rainfall, log			−0.008	−0.005	−0.014
			(0.117)	(0.116)	(0.115)
School closure					−0.285
					(0.189)
Gathering restriction					0.263*
					(0.111)
Prefecture FE	X	X	X	X	X
Week FE	X	X	X	X	X
Observations	402	402	402	402	402
R^2	0.932	0.932	0.932	0.932	0.933
Adjusted R^2	0.916	0.916	0.916	0.916	0.916

Notes: *p<0.1; **p<0.05; ***p<0.01 The dependent variable is the log-transformed value of the number of new infection cases (1 week lag). The unit of analysis is prefecture and week, and the fixed effects are introduced in all models. For the observations, six prefectures are targeted, and the period of analysis is for 67 weeks from the third week of January, 2020 to the fourth week of April, 2021. The values in parentheses are cluster-robust standard errors. Clustering is at the prefecture level. Cumulative GZ-certified restaurants, log is the log-transformed value of the number of cumulative certified-GZ restaurants. Cumulative GZ-certified restaurants and hotels, log is the log-transformed value of the number of cumulative certified-GZ restaurants and hotels. Infectious, log is the log-transformed value of the number of potentially infected people. Susceptible, log is the log-transformed value of the total number of susceptible population. State of Emergency is the dummy variable that takes the value 1 if the state of emergency is declared. Tests (1 week lag), log is the log-transformed value of the number of COVID-19 tests. Customers per restaurant, log is the log-transformed value of the number of customers per restaurant. Average temperature, log is the log-transformed value of the mean temperature (Fahrenheit degrees). Average rainfall, log is the log-transformed value of the aggregated rainfall (in millimeters). School closure is the dummy variable that takes the value 1 if the school closure is declared. Gathering restriction is the dummy variable that takes the value 1 if the large-scale gathering restriction is declared. For the variables that take absolute value 0 (New infection cases (1 week lag), log, Cumulative GZ-certified restaurants, log, Cumulative GZ-certified restaurants and hotels, log, Infectious, log, and Tests (1 week lag), log), we add value 1 before log-transforming to avoid the logarithm of 0.

D.3 Economic effects (restaurants' sales and the number of customers)

Table 8: Restaurants' sales and customers (POS) and the Green Zone certification

	Dependent variable:							
	Sales per restaurant, log				Customers per restaurant, log			
	(1)	(2)	(3)	(4)	(5)	(6)	(7)	(8)
Cumulative GZ-certified restaurants, log	0.018***	0.016***	0.016***	0.016***	0.040***	0.037***	0.037***	0.037***
	(0.002)	(0.003)	(0.004)	(0.003)	(0.003)	(0.005)	(0.005)	(0.005)
State of Emergency	−0.267***	−0.264***	−0.257***	−0.261***	−0.223***	−0.219***	−0.217***	−0.219***
	(0.018)	(0.017)	(0.014)	(0.037)	(0.032)	(0.030)	(0.030)	(0.040)
The number of new COVID-19 cases, log		−0.013	−0.015	−0.015		−0.017	−0.018	−0.018
		(0.010)	(0.011)	(0.011)		(0.012)	(0.013)	(0.012)
Average temperature, log			0.296*	0.298*			0.188*	0.187*
			(0.129)	(0.128)			(0.089)	(0.091)
Average rainfall, log			−0.007	−0.007			−0.012**	−0.012**
			(0.005)	(0.005)			(0.004)	(0.004)
School closure				0.085				−0.062
				(0.108)				(0.072)
Gathering restriction				0.004				0.002
				(0.041)				(0.035)
Prefecture FE	X	X	X	X	X	X	X	X
Day FE	X	X	X	X	X	X	X	X
Observations	5,106	5,106	5,106	5,106	5,106	5,106	5,106	5,106
R^2	0.935	0.935	0.935	0.935	0.947	0.947	0.947	0.947
Adjusted R^2	0.921	0.922	0.922	0.922	0.936	0.936	0.937	0.937

Notes: *p<0.1; **p<0.05; ***p<0.01 The dependent variable is the log-transformed value of POS sales per restaurant and the number of customers per restaurant. The unit of analysis is prefecture and day, and the fixed effects are introduced in all models. For the observations, six prefectures are targeted, and the period of analysis is for 912 days from January 1st, 2019 to April 30th, 2021. The values in parentheses are cluster-robust standard errors. Clustering is at the prefecture level. Cumulative GZ-certified restaurants, log is the log-transformed value of the number of cumulative certified-GZ restaurants. State of Emergency is the dummy variable that takes the value 1 if the state of emergency is declared. The number of new COVID-19 cases, log is the log-transformed value of the daily number of infection cases. Average temperature, log is the log-transformed value of the mean temperature (Fahrenheit degrees). Average rainfall, log is the log-transformed value of the aggregated rainfall (in millimeters). School closure is the dummy variable that takes the value 1 if the school closure is declared. Gathering restriction is the dummy variable that takes the value 1 if the large-scale gathering restriction is declared. For the variables that take absolute value 0 (Cumulative GZ-certified restaurants, log, and The number of new COVID-19 cases, log), we add value 1 before log-transforming to avoid the logarithm of 0.

D.4 Restaurant information views online

Table 9: Restaurant information views online (percentage change) and the Green Zone certification

	Dependent variable:		
	Restaurant information views online (percentage change)		
	(1)	(2)	(3)
Cumulative GZ-certified restaurants, log	1.900***	1.497***	1.297***
	(0.252)	(0.253)	(0.308)
State of Emergency	−3.842**	−4.281**	−8.499**
	(1.164)	(1.390)	(2.792)
The number of new COVID-19 cases, log		−1.709**	−1.984***
		(0.449)	(0.466)
Average temperature, log		−25.388	−24.386
		(17.074)	(17.322)
Average rainfall, log		−0.372	−0.517
		(0.480)	(0.512)
School closure			6.563**
			(2.242)
Gathering restriction			4.808
			(4.042)
The mean of dep. variable in Yamanashi Prefecture		-21.052	
The mean of dep. variable in the control group		-22.641	
Prefecture FE	X	X	X
Week FE	X	X	X
Observations	408	408	408
R^2	0.947	0.950	0.953
Adjusted R^2	0.935	0.939	0.941

Notes: *p<0.1; **p<0.05; ***p<0.01 The dependent variable is the percentage change in the number of restaurant information views online compared to the 2019 baseline (V-RESAS). The unit of analysis is prefecture-week, and the fixed effects are introduced in all models. For the observations, six prefectures are targeted, and the period of analysis is for 68 weeks from the third week of January, 2020 to the fifth week of April, 2021. The values in parentheses are cluster-robust standard errors. Clustering is at the prefecture level. Cumulative GZ-certified restaurants, log is the log-transformed value of the number of cumulative certified-GZ restaurants. State of Emergency is the dummy variable that takes the value 1 if the state of emergency is declared. The number of new COVID-19 cases, log is the log-transformed value of the daily number of infection cases. Average temperature, log is the log-transformed value of the mean temperature (Fahrenheit degrees). Average rainfall, log is the log-transformed value of the aggregated rainfall (in millimeters). School closure is the dummy variable that takes the value 1 if the school closure is declared. Gathering restriction is the dummy variable that takes the value 1 if the large-scale gathering restriction is declared. For the variables that take absolute value 0 (Cumulative GZ-certified restaurants, log, and The number of new COVID-19 cases, log), we add value 1 before log-transforming to avoid the logarithm of 0.

D.5 Human mobility by facility type

Table 10: Human mobility by facility type and the Green Zone certification

	Dependent variable:											
	retail/recreation		grocery/pharmacy		parks		transit stations		workplaces		residential	
	(1)	(2)	(3)	(4)	(5)	(6)	(7)	(8)	(9)	(10)	(11)	(12)
Cumulative GZ-certified restaurants, log	0.372***	0.366***	0.051	0.099	2.804***	3.138***	0.448***	0.548**	−0.029	−0.003	−0.061***	−0.060***
	(0.042)	(0.045)	(0.038)	(0.062)	(0.101)	(0.346)	(0.051)	(0.180)	(0.051)	(0.048)	(0.013)	(0.006)
State of Emergency	−3.992***	−3.890***	−0.245	−0.393	4.231	5.191	−2.007	−1.387	−1.410***	−1.282***	1.006***	0.964***
	(0.583)	(0.421)	(0.468)	(0.357)	(4.044)	(3.188)	(2.514)	(2.132)	(0.343)	(0.258)	(0.234)	(0.171)
The number of new COVID-19 cases, log		−0.180		0.121		0.538		0.127		0.031		0.060*
		(0.107)		(0.129)		(0.972)		(0.527)		(0.070)		(0.029)
Average temperature, log		8.489**		5.912**		74.310***		27.060**		6.769**		−3.482**
		(2.193)		(1.679)		(7.775)		(7.974)		(1.881)		(0.989)
Average rainfall, log		−0.769***		−1.084***		−4.338***		−0.653		−0.261***		0.298***
		(0.101)		(0.088)		(0.520)		(0.329)		(0.061)		(0.031)
Prefecture FE	X	X	X	X	X	X	X	X	X	X	X	X
Date FE	X	X	X	X	X	X	X	X	X	X	X	X
Observations	2,646	2,646	2,646	2,646	2,627	2,627	2,646	2,646	2,646	2,646	2,646	2,646
R^2	0.965	0.967	0.897	0.910	0.837	0.860	0.899	0.905	0.991	0.991	0.984	0.987
Adjusted R^2	0.958	0.961	0.876	0.891	0.803	0.830	0.879	0.885	0.989	0.989	0.981	0.984

Notes: *p<0.1; **p<0.05; ***p<0.01 The dependent variable is the percent change in human flow for a given facility type compared to the January 2020 baseline (Google Mobility). The unit of analysis is prefecture and day, and the fixed effects are introduced in all models. For the observations, six prefectures are targeted, and the period of analysis is for 441 days from February 15th, 2020 to April 30, 2021. The values in parentheses are cluster-robust standard errors. Clustering is at the prefecture level. Cumulative GZ-certified restaurants, log is the log-transformed value of the number of cumulative certified-GZ restaurants. State of Emergency is the dummy variable that takes the value 1 if the state of emergency is declared. The number of new COVID-19 cases, log is the log-transformed value of the daily number of infection cases. Average temperature, log is the log-transformed value of the mean temperature (Fahrenheit degrees). Average rainfall, log is the log-transformed value of the aggregated rainfall (in millimeters). School closure is the dummy variable that takes the value 1 if the school closure is declared. Gathering restriction is the dummy variable that takes the value 1 if the large-scale gathering restriction is declared. For the variables that take absolute value 0 (Cumulative GZ-certified restaurants, log, and The number of new COVID-19 cases, log), we add value 1 before log-transforming to avoid the logarithm of 0.

D.6 Inter-regional Mobility

Table 11: Inter-regional mobility and the Green Zone certification

	Dependent variable:								
	intracity			intercity			interprefectural		
	(1)	(2)	(3)	(4)	(5)	(6)	(7)	(8)	(9)
Cumulative GZ-certified restaurants, log	-0.115^{***}	-0.106^{***}	-0.109^{***}	0.224^{**}	0.177^{*}	0.204^{*}	1.055^{***}	1.132^{***}	0.788^{***}
	(0.022)	(0.026)	(0.024)	(0.060)	(0.081)	(0.083)	(0.163)	(0.148)	(0.148)
State of Emergency	0.630^{**}	0.589^{**}	0.533^{**}	-5.249^{***}	-5.128^{***}	-4.586^{***}	-0.125	-0.212	-7.466^{*}
	(0.240)	(0.166)	(0.190)	(0.585)	(0.459)	(0.589)	(2.457)	(2.680)	(2.932)
The number of new COVID-19 cases, log		0.050	0.046		-0.254	-0.220		0.424	-0.050
		(0.050)	(0.047)		(0.177)	(0.178)		(0.466)	(0.371)
Average temperature, log		-1.461	-1.465		6.131	6.090		-10.450	-8.691
		(1.701)	(1.705)		(3.778)	(3.707)		(16.018)	(16.521)
Average rainfall, log		0.007	0.005		-0.167	-0.149		0.552	0.303
		(0.107)	(0.108)		(0.317)	(0.319)		(0.735)	(0.743)
School closure			-0.124			0.231			11.736^{***}
			(0.184)			(0.850)			(1.441)
Gathering restriction			0.062			-0.611			8.271^{**}
			(0.145)			(0.354)			(3.190)
Mean of Mobility in Yamanashi		5.49			-7.079			-27.778	
Mean of Mobility in Control		5.165			-7.584			-27.881	
Prefecture FE	X	X	X	X	X	X	X	X	X
Week FE	X	X	X	X	X	X	X	X	X
Observations	408	408	408	408	408	408	408	408	408
R^2	0.980	0.980	0.980	0.963	0.963	0.963	0.942	0.943	0.949
Adjusted R^2	0.975	0.975	0.975	0.954	0.955	0.955	0.929	0.929	0.936

Notes: *p<0.1; **p<0.05; ***p<0.01 The dependent variable is the percentage change in inter-regional human flow (within a city, within a prefecture, and across prefectures) compared to the 2019 baseline (V-RESAS). The unit of analysis is prefecture and week, and the fixed effects are introduced in all models. For the observations, six prefectures are targeted, and the period of analysis is for 68 weeks from the third week of January, 2020 to the fifth week of April, 2021. The values in parentheses are cluster-robust standard errors. Clustering is at the prefecture level. Cumulative GZ-certified restaurants, log is the log-transformed value of the number of cumulative certified-GZ restaurants. State of Emergency is the dummy variable that takes the value 1 if the state of emergency is declared. The number of new COVID-19 cases, log is the log-transformed value of the daily number of infection cases. Average temperature, log is the log-transformed value of the mean temperature (Fahrenheit degrees). Average rainfall, log is the log-transformed value of the aggregated rainfall (in millimeters). School closure is the dummy variable that takes the value 1 if the school closure is declared. Gathering restriction is the dummy variable that takes the value 1 if the large-scale gathering restriction is declared. For the variables that take absolute value 0 (Cumulative GZ-certified restaurants, log, and The number of new COVID-19 cases, log), we add value 1 before log-transforming to avoid the logarithm of 0.

D.7 Stay-home rate

Table 12: The stay-home rate by male age group and the Green Zone certification

	Dependent variable:						
	Male 15-19 y/o	Male 20s	Male 30s	Male 40s	Male 50s	Male 60s	Male 70s
	(1)	(2)	(3)	(4)	(5)	(6)	(7)
Cumulative GZ-certified restaurants, log	0.003**	−0.002**	−0.003***	−0.002***	−0.002***	−0.003*	−0.003**
	(0.001)	(0.001)	(0.000)	(0.000)	(0.000)	(0.001)	(0.001)
State of Emergency	0.003	0.011**	0.020***	0.013***	0.011***	0.028***	−0.018*
	(0.016)	(0.003)	(0.003)	(0.003)	(0.002)	(0.006)	(0.008)
The number of new COVID-19 cases, log	0.001	0.004**	0.003*	0.002*	0.002*	0.000	0.005**
	(0.003)	(0.001)	(0.001)	(0.001)	(0.001)	(0.001)	(0.002)
Average temperature, log	0.027	−0.039	−0.027	−0.013	−0.015	−0.084	−0.128*
	(0.050)	(0.024)	(0.039)	(0.039)	(0.027)	(0.048)	(0.062)
Average rainfall, log	0.009*	0.002*	0.004***	0.006***	0.006***	0.008***	0.015***
	(0.004)	(0.001)	(0.000)	(0.001)	(0.001)	(0.001)	(0.002)
School closure	0.007	0.019*	0.017**	0.007	0.012**	0.024	0.024
	(0.050)	(0.008)	(0.005)	(0.005)	(0.004)	(0.015)	(0.013)
Gathering restriction	0.040**	0.005	0.014***	0.011***	0.011***	0.012**	0.021***
	(0.013)	(0.004)	(0.003)	(0.002)	(0.002)	(0.003)	(0.004)
Prefecture FE	X	X	X	X	X	X	X
Day FE	X	X	X	X	X	X	X
Observations	2,694	2,694	2,694	2,694	2,694	2,694	2,694
R^2	0.925	0.931	0.950	0.964	0.964	0.959	0.947
Adjusted R^2	0.910	0.917	0.940	0.956	0.957	0.951	0.936

Notes: *p<0.1; **p<0.05; ***p<0.01 The dependent variable is the day-time (from 9 am to 6 pm) stay-home rate for males, which indicates the percentage of people who refrain from going out compared to the baseline value; the closer to 1, the more people refrain from going out, and the closer to 0, the more people go out. The unit of analysis is prefecture and day, and the fixed effects are introduced in all models. For the observations, six prefectures are targeted, and the period of analysis is for 449 days from January 1st, 2020 to March 24th, 2021. The values in parentheses are cluster-robust standard errors. Clustering is at the prefecture level. Cumulative GZ-certified restaurants, log is the log-transformed value of the number of cumulative certified-GZ restaurants. State of Emergency is the dummy variable that takes the value 1 if the state of emergency is declared. The number of new COVID-19 cases, log is the log-transformed value of the daily number of infection cases. Average temperature, log is the log-transformed value of the mean temperature (Fahrenheit degrees). Average rainfall, log is the log-transformed value of the aggregated rainfall (in millimeters). School closure is the dummy variable that takes the value 1 if the school closure is declared. Gathering restriction is the dummy variable that takes the value 1 if the large-scale gathering restriction is declared. For the variables that take absolute value 0 (Cumulative GZ-certified restaurants, log, and The number of new COVID-19 cases, log), we add value 1 before log-transforming to avoid the logarithm of 0.

Table 13: The stay-home rate by female age group and the Green Zone certification

	Dependent variable:						
	Female 15-19 y/o	Female 20s	Female 30s	Female 40s	Female 50s	Female 60s	Female 70s
	(1)	(2)	(3)	(4)	(5)	(6)	(7)
Cumulative GZ-certified restaurants, log	−0.002	−0.002	−0.003**	−0.002***	−0.001*	−0.002***	−0.003**
	(0.001)	(0.001)	(0.001)	(0.000)	(0.001)	(0.000)	(0.001)
State of Emergency	−0.009	0.009	0.011	0.012**	0.020***	0.019***	0.023**
	(0.015)	(0.006)	(0.006)	(0.004)	(0.005)	(0.003)	(0.007)
The number of new COVID-19 cases, log	0.002	0.002	0.003	0.002	0.002	0.003**	0.004*
	(0.002)	(0.002)	(0.002)	(0.001)	(0.001)	(0.001)	(0.002)
Average temperature, log	0.006	−0.063	−0.024	0.006	−0.022	−0.032	−0.136*
	(0.062)	(0.038)	(0.051)	(0.029)	(0.028)	(0.043)	(0.057)
Average rainfall, log	0.006*	0.004***	0.006***	0.006***	0.006***	0.007***	0.012***
	(0.003)	(0.001)	(0.001)	(0.001)	(0.001)	(0.001)	(0.001)
School closure	0.007	0.025	0.016	0.006	0.015**	0.009	0.012
	(0.052)	(0.016)	(0.010)	(0.014)	(0.005)	(0.016)	(0.012)
Gathering restriction	0.048***	0.005	0.014***	0.014***	0.009***	0.009***	0.019***
	(0.011)	(0.004)	(0.003)	(0.002)	(0.002)	(0.002)	(0.004)
Prefecture FE	X	X	X	X	X	X	X
Day FE	X	X	X	X	X	X	X
Observations	2,694	2,694	2,694	2,694	2,694	2,694	2,694
R^2	0.936	0.943	0.960	0.966	0.966	0.961	0.948
Adjusted R^2	0.922	0.931	0.951	0.959	0.959	0.953	0.938

Notes: *p<0.1; **p<0.05; ***p<0.01 The dependent variable is the day-time (from 9 am to 6 pm) stay-home rate for females, which indicates the percentage of people who refrain from going out compared to the baseline value; the closer to 1, the more people refrain from going out, and the closer to 0, the more people go out. The unit of analysis is prefecture and day, and the fixed effects are introduced in all models. For the observations, six prefectures are targeted, and the period of analysis is for 449 days from January 1st, 2020 to March 24th, 2021. The values in parentheses are cluster-robust standard errors. Clustering is at the prefecture level. Cumulative GZ-certified restaurants, log is the log-transformed value of the number of cumulative certified-GZ restaurants. State of Emergency is the dummy variable that takes the value 1 if the state of emergency is declared. The number of new COVID-19 cases, log is the log-transformed value of the daily number of infection cases. Average temperature, log is the log-transformed value of the mean temperature (Fahrenheit degrees). Average rainfall, log is the log-transformed value of the aggregated rainfall (in millimeters). School closure is the dummy variable that takes the value 1 if the school closure is declared. Gathering restriction is the dummy variable that takes the value 1 if the large-scale gathering restriction is declared. For the variables that take absolute value 0 (Cumulative GZ-certified restaurants, log, and The number of new COVID-19 cases, log), we add value 1 before log-transforming to avoid the logarithm of 0.

Table 14: The night-time stay-home rate and the Green Zone certification

	Dependent variable:		
	Night-time stay-home rate		
	(1)	(2)	(3)
Cumulative GZ-certified restaurants, log	-0.004^{***}	-0.003^{**}	-0.004^{***}
	(0.001)	(0.001)	(0.001)
State of Emergency	0.086^{***}	0.088^{***}	0.070^{***}
	(0.014)	(0.015)	(0.017)
The number of new COVID-19 cases, log		0.005	0.004
		(0.003)	(0.003)
Average temperature, log		0.023	0.024
		(0.031)	(0.032)
Average rainfall, log		0.009^{***}	0.009^{***}
		(0.002)	(0.002)
School closure			-0.001
			(0.004)
Gathering restriction			0.021^{*}
			(0.010)
Prefecture FE	X	X	X
Day FE	X	X	X
Observations	2,724	2,724	2,724
R^2	0.947	0.948	0.948
Adjusted R^2	0.937	0.937	0.938

Notes: *p<0.1; **p<0.05; ***p<0.01 The dependent variable is the night-time (from 8pm to 0am) stay-home rate, which indicates the percentage of people who refrain from going out compared to the baseline value; the closer to 1, the more people refrain from going out, and the closer to 0, the more people go out. The unit of analysis is prefecture and day, and the fixed effects are introduced in all models. For the observations, six prefectures are targeted, and the period of analysis is for 454 days from January 1st, 2020 to March 29th, 2021. The values in parentheses are cluster-robust standard errors. Clustering is at the prefecture level. Cumulative GZ-certified restaurants, log is the log-transformed value of the number of cumulative certified-GZ restaurants. State of Emergency is the dummy variable that takes the value 1 if the state of emergency is declared. The number of new COVID-19 cases, log is the log-transformed value of the daily number of infection cases. Average temperature, log is the log-transformed value of the mean temperature (Fahrenheit degrees). Average rainfall, log is the log-transformed value of the aggregated rainfall (in millimeters). School closure is the dummy variable that takes the value 1 if the school closure is declared. Gathering restriction is the dummy variable that takes the value 1 if the large-scale gathering restriction is declared. For the variables that take absolute value 0 (Cumulative GZ-certified restaurants, log, and The number of new COVID-19 cases, log), we add value 1 before log-transforming to avoid the logarithm of 0.

E Treatment Effect

E.1 Comparison of Treatment and Control prefecture

	Yamanashi	Shizuoka	Tochigi	Nagano	Gunma	Ibaraki
Population (in thousands)	811	3,644	2,049	1,942	1,934	2,860
Population density ($/km^2$) *	4,668	5,267	4,244	3,997	4,691	4,570
Distance to Tokyo (km)	101.7	142.8	172.8	96.4	98.8	99.3

Notes: Population and population density are from the 2019 and 2014 National Census, respectively. For distance to Tokyo, see "Distance between Prefectural Offices" by the Geospatial Information Authority of Japan. *In prefectures in the Tokyo metropolitan area that are excluded from the control group, population density is about two to three times that of Yamanashi. In particular, the population densities of Tokyo, Kanagawa, Saitama, and Chiba prefectures are 12,022, 8,979, 8,340, and 7,145 persons/km^2, respectively.

E.2 Comparison of policies in Treatment and Control prefecture

	Yamanashi	Shizuoka	Tochigi	Nagano	Gunma	Ibaraki
Title of the certification policy	Yamanashi Green Zone Certification	Fujinokuni Safety and Security Certification	Tochimaru Reliable Certification	Shinshu Safe Store Certification System	Stop Covid-19! Countermeasure Certification System	Ibaraki's Amabie-chan
Introduction date	May 2020	May 2021	May 2021	April 2021	July 2020	June 2020
Third-party onsite inspection requirements	Yes	Yes	Yes	Yes	Yes	Yes (from April 14, 2021)
Subsidies on indoor infection control measures	From July 10, 2020	Only in Hamamatsu City	From January 22, 2021	From September 15, 2020 – December 28, 2020	Only in Maebashi City	From October 2, 2020 to December 31, 2020
Prefectural/municipal support on the introduction of delivery services	In certain cities	From April 17, 2020	From January 22, 2021	From September 15, 2020 – December 28, 2020	Only in Maebashi City	In certain cities

Notes: Prepared by the authors with reference to prefectures' press releases and newspaper articles. Gunma Prefecture introduced a certification system around the same time as Yamanashi, but the penetration rate remains 21.6% as of October 2020 (see Reference List 17–35).

E.3 List of Business suspension request

	First Emergency Declaration (April 2020 – May 2020)	Second Emergency Declaration (January 2021 – March 2021)	Third Emergency Declaration (April 2021 – June 2021)
Yamanashi	April 20 – May 14 (business closure on bars and nightclubs) May 15 – February 12 (qualified facilities were individually exempted)	January 25 – February 7	No
Shizuoka	April 25 – May 17 (bars and nightclubs only)	December 23 – January 5 (only in Fuji City)	May 19 – June 1 (only in Kosai City)
Tochigi	April 18 – May 15 (restriction on alcohol service hours only)	January 8 – February 21 (in specified cities until January 12)	No
Nagano	April 23 – May 15	January 18 – February 4 (in specified cities)	April 2 – 9 (Nagano City) April 21 – 29 (in specified cities)
Gunma	April 18 – May 15	December 15 – March 1 (in specified cities)	May 8 – June 20
Ibaraki	April 18 – May 17 (bars and nightclubs only)	November 30 – December 20, January 6 – 17 (in specified cities) January 18 – February 22 (entire Prefecture)	April 22 – June 16 (in specified cities)

Notes: Prepared by the authors with reference to each prefecture's official website and newspapers (see Reference List 36–64).

E.4 Yamanashi Green Zone Certification criteria

Standards pertaining to measures for prevention of infectious diseases (Restaurant industry)

E.4.1 Prevention of infectious diseases among visitors

(1) Store entry, order, and payment

- Disinfection equipment shall be installed at the entrance of the store, and hand sanitization shall be indicated at the entrance.
- When there is a queue due to waiting for a turn, etc., a minimum distance of 1 meter (2 meters if no mask is worn) shall be maintained between visitors.
- When serving customers face-to-face at the cash register, etc., use acrylic panels, transparent vinyl curtains, partitions, etc. to shield the customers. In addition, use coin trays or introduce cashless payment.
- Those with fever (e.g., 1 degree above normal), cold symptoms (e.g., cough, sore throat), vomiting, diarrhea, etc., even if they have mild symptoms, should not be admitted.
- Make it known that people should wear masks except when eating or drinking, and request that people wash their hands and disinfect their hands regularly.
- Remind people to practice good cough etiquette.
- If there is an elevator, limit the number of passengers by adjusting the weight sensor of the elevator. Capacity:______, Passenger limit:____
- If there is a pick-up truck, shield the driver's seat and rear seat of the pick-up truck with an acrylic plate or transparent vinyl curtain.

(2) Meals and in-store use

[One of these must be met for placement between tables]

- Tables used by the same group and tables used by other groups should be placed so that there is at least 1 meter of interpersonal distance between them.
  - Table-to-table distance: _____ m
- Use acrylic panels, transparent plastic curtains, partitions, etc. to shield the space between tables used by the same group and tables used by other groups.

[One of the following conditions must be met for placement on the same table]

Exclude cases where a small number of family members, elderly people with caregivers, infants, disabled people, etc. wish to sit face-to-face.

- Do not place seating directly in front of each other. Seating should be arranged so that the distance between seats is at least 1 meter. Seat-to-seat distance: _____m.
- Install partitions on tables to shield them.

- Avoid having too many people at the same time by limiting the length of stay* and using a reservation system. (*Approximately 2 hours).

- Avoid large plates and serve food individually, or have employees serve the food.

[In buffet style, one of the following must be met]

- A new small plate should be used by each user for each serving, and food and drinks should be protected by covers to prevent splashing, and masks, disposable gloves, etc. should be worn when serving. When serving, make sure to wear masks, disposable gloves, etc., and do not share tongs or chopsticks for serving.
- Serve food on small plates or have staff serve food.

- Avoid setting up common tabletop condiments, pots, etc., or disinfect them when changing customers.
- Remind customers not to share or use spoons, chopsticks, or other utensils.
- Reduce the volume of background music in the store and remind customers to avoid loud conversations.
- Coughing etiquette should be strictly observed. (For ventilation standards, see "3. Hygiene Management of Facilities and Equipment" for ventilation standards).
- If the toilet has a lid, indicate that waste should be flushed after the lid is closed.
- Indicate that people should wash their hands and disinfect their hands after using the restroom.
- If there is a smoking area, reduce the number of people using it at one time, and keep a distance between people. If there is a smoking area, request that the three densities be avoided by reducing the number of people using the area at once, keeping a good distance between people, etc.
  - Size of the smoking space: ________ m^2 Maximum capacity: ________

E.4.2 Prevention of infectious diseases among employees

- Make sure to wear masks.
- Take your temperature and check your physical condition before starting work.
- If there are multiple rooms, limit per smoking space. If you have a fever (e.g., more than 1 degree above normal), a cold (cough, sore throat, etc.), vomiting, diarrhea, or other symptoms, even if they are mild. symptoms such as vomiting or diarrhea.
- Employees who are infected or suspected to be infected, or who are judged to be in close contact with infected employees, are prohibited from working. Employees who are infected, suspected to be infected, or determined to be a close contact shall not be allowed to work.
- Hand disinfection and hand washing are to be performed regularly at the beginning of work, after touching areas or items that come into contact with others, after cleaning, and after using the toilet.
- When accepting orders from users or serving food, be careful not to stand in front of users and maintain a safe distance from them.
- In the break area, reduce the number of people taking a break at one time, and avoid eating and talking face-to-face.
- Ventilate the break area at all times (for ventilation standards, refer to "3. Thorough hygiene management of facilities and equipment") and disinfect shared items on a regular basis.
- Employees' uniforms should be laundered regularly after work on the day in question.
  - Frequency of uniform washing: ____________

E.4.3 Thorough hygiene management of facilities and equipment

- For facilities subject to the Building Management Law*, check whether they meet the standards for air quality control based on the law, and if not, maintain and manage the ventilation equipment appropriately, including cleaning and maintenance.
  - * Law Concerning the Protection of Sanitary Environments in Buildings

---

[For facilities not covered by the Building Management Law, one of the following must be met]

- The required ventilation volume (30 m^3 per hour per person) shall be secured by ventilation equipment. If the required ventilation volume is not enough, the ventilation system shall be installed.
- If the required ventilation volume is not sufficient, adjust the number of people entering the store to secure the required ventilation volume per person, and properly maintain the ventilation equipment, including cleaning and maintenance.
- For ventilation by opening windows, open all windows in two directions (or open the door if there is only one window) once every 30 minutes for about 5 minutes to ensure sufficient ventilation.

[Appeal items] This is not a mandatory requirement for certification, but it is an item that can be appealed as a voluntary effort by the business.

- The details of ventilation (air flow) in common areas where people are crowded in the facility are clearly shown.
- In order to secure the required amount of ventilation per person in a densely populated common area in the facility, the ventilation system should be designed for each area.
  - (In the case of limiting the number of persons to ensure the required ventilation volume) Ventilation volume: m^3/hour ÷ 30 m^3/person/hour = ____person

---

- Prohibit the use of hand dryers and common towels, and provide paper towels or encourage the use of personal towels.
- Wipe down and disinfect items shared with others and areas that are touched by multiple people regularly, such as when changing users, using disinfecting ethanol, sodium hypochlorite, or commercially available detergents containing surfactants.
  - [areas shared with others in the restaurant industry and frequently touched]
    - Tables, chairs, menu books, condiments, drink bars, doorknobs, light switches, touch panels, tabletop bells, cash registers, faucets, handrails, toilet seats, washing levers, coin trays, ticket vending machines, elevator buttons, etc.

[Appeal items]

- In order to reduce the risk of contact and droplet infections, the following measures should be taken to avoid overlapping lines of flow for users. Describe in detail: ____________________

- Those who collect garbage should wear masks and gloves, and always wash their hands after work.
- Garbage, hand towels, etc. that may have food residue, snot, saliva, etc. on them should be sealed in plastic bags. sealed in a plastic bag for disposal.

E.4.4 Preparation and publication of checklists

- Each facility or business operator shall prepare a checklist that specifies specific methods and procedures, frequency of cleaning and disinfection, spacing between people, etc., after assessing the risks in the facility, and disclose the daily checks using the checklist.

E.4.5 Policy for dealing with an outbreak of infection

- In the event that an employee of the facility is found to be infected, the facility will respond and cooperate with the public health center's instructions and investigations in a sincere and proactive manner, take measures to prevent the spread of infection from the facility, and if necessary, publicize information to prevent the spread of infection, such as business days when there is a possibility of infection.
- Provide employees with training opportunities to ensure that they are taking appropriate actions to prevent the spread of infection, such as refraining from going to work if they are suspected of being infected until the test results are known.
- If the results of a proactive epidemiological survey conducted by the public health center reveal that an infected person has been using the facility in question, take measures to prevent the spread of infection through the facility in question by responding and cooperating with the public health center's advice and instructions in good faith and proactively.

[Appeal Items]

- In order to identify the risk of infection at an early stage, employees should be encouraged or required to use an application for notification of close contact provided by the government.
- In addition to the above, introduce a system for early identification of infection risk.

Describe in detail: ______________________________

References

Academic Article

Law, K.B., Peariasamy, K.M., Gill, B.S. et al. Tracking the early depleting transmission dynamics of COVID-19 with a time-varying SIR model. Sci Rep 10, 21721 (2020). https://doi.org/10.1038/s41598-020-78739-8

Information by Media & Public Institutions

1. Tottori Prefecture. Progress of the government's response. Special site for novel coronavirus infections. https://www.pref.tottori.lg.jp/289733.htm. (2021).
2. NHK. Record of all COVID-19 related articles. Special site for novel coronavirus infections. https://www3.nhk.or.jp/news/special/coronavirus/chronology/?mode=all&target=202003. (2021).

3. Yamanashi Prefecture. Statistical information on new coronavirus infections (outbreaks, testing status, number of consultations). https://www.pref.yamanashi.jp/koucho/coronavirus/info_coronavirus_emergencymeasures18.html. (2021).
4. Shizuoka Prefecture. Shizuoka Prefecture Open Data: Trends of new coronavirus infections in the prefecture. https://opendata.pref.shizuoka.jp/dataset/8167.html. (2022).
5. Nagano Prefecture. Trends of novel coronavirus infections in Nagano Prefecture. https://www.pref.nagano.lg.jp/hoken-shippei/kenko/kenko/kansensho/joho/corona-doko.html. (2022).
6. Gunma Prefecture. Latest trends in the prefecture. https://stopcovid19.pref.gunma.jp/. (2022).
7. Tochigi Prefecture. Outbreak and Testing Status of Novel Coronavirus Infections in Tochigi Prefecture. https://a01sa01to.com/opendata/covid19_ibaraki/test_people. (2022).
8. Asa. New Coronavirus Infections in Ibaraki Prefecture: Number of people tested (Prefectural Institute of Public Health and Mito City Public Health Center). https://a01sa01to.com/opendata/covid19_ibaraki/test_people. (2022).
9. Cabinet Office, Government of Japan. V-RESAS. https://v-resas.go.jp/. (2021).
10. Agoop Corp. Population flow data. https://www.agoop.co.jp/service/dynamic-population-data/. (2021).
11. Google. COVID-19 community mobility reports. https://www.google.com/covid19/mobility/. (2020).
12. Japan Meteorological Agency. Past weather data search. https://www.data.jma.go.jp/obd/stats/etrn/. (2022).
13. Mizuno Laboratory. COVID-19 special site: Visualizing the stay-home rate. National Institute of Informatics, Sendai. http://research.nii.ac.jp/~mizuno/covid19.html. (2022).
14. Japan Statistics Bureau. 2014 Census. (2014).
15. Japan Statistics Bureau. 2019 Census. (2019).
16. Geospatial Information Authority of Japan. Distances between prefectural offices. https://www.gsi.go.jp/common/000195510.pdf. (2018).
17. Nihon Keizai Shimbun. Yamanashi tops the list with 98% of restaurants certified; 7 prefectures have less than 10%. https://www.nikkei.com/article/DGXZQOCC144570U1A011C2000000/. (2021).
18. Yamanashi Prefecture. Applying for certification. https://greenzone-ninsho.jp/apply/index.html. (2021).
19. Hojokin Portal. Yamanashi Prefecture: Support for purchasing equipment to promote new lifestyles. https://hojyokin-portal.jp/subsidies/7115. (2021).
20. Toru City. Grant to support the catering industry in Toru City. https://www.city.tsuru.yamanashi.jp/soshiki/sangyo/shoko_t/6/11538.html. (2021).
21. Kofu City. Ganbarou, Kofu! Delivery support grant (Kofu City food delivery service introduction support grant). https://www.city.kofu.yamanashi.jp/shoko/kofutakuhai/takuhai.html. (2021).
22. Shizuoka Prefecture. Subsidy for projects to promote the Fuji-no-Kuni Safety and Security Certification System (restaurants). http://www.pref.shizuoka.jp/kinkyu/covid-19-anzen_anshin_ninsyo_hozyo.html. (2021).
23. Nihon Keizai Shimbun. Novel coronavirus disease, Shizuoka Prefecture to subsidize small and medium-sized businesses for home delivery. https://www.nikkei.com/article/DGXMZO58203360X10C20A4L61000/. (2020).
24. Tochigi Prefecture. Tochigi Prefecture subsidy for supporting measures against infectious diseases at local companies. https://www.tochigi-kansentaisaku.com/. (2021).
25. Nagano City. About subsidies for businesses supporting measures against the novel coronavirus. https://www.city.nagano.nagano.jp/site/covid19-joho/456721.html. (2021).

26. Nagano Prefecture. Droplet prevention panels (acrylic panels) will be distributed free of charge to restaurants. https://www.city.nagano.nagano.jp/site/covid19-joho/456721.html. (2021).
27. Nagano Prefecture. About the "Support Project for Countermeasures against the Novel Coronavirus in the Restaurant and Service Industry." https://www.pref.nagano.lg.jp/eigyo/ouenhojokin.html. (2021).
28. Nagano City. Subsidies for businesses supporting measures against the novel coronavirus. https://www.city.nagano.nagano.jp/site/covid19-joho/456721.html. (2021).
29. Maebashi City. Registration Closed FY2021 Maebashi City New Normal Support Subsidy. https://www.city.maebashi.gunma.jp/soshiki/sangyokeizai/nigiwaishogyo/shinseisho/30394.html. (2020).
30. Itakura Town. FY2021 2nd Stop Corona in Itakura Town! Incentives for stores certified to take countermeasures. http://www.town.itakura.gunma.jp/cont/s021000/d021010/20201215141115.html. (2021).
31. Nihon Keizai Shimbun. City subsidizes lunch box delivery by cab in Maebashi, taking advantage of deregulation. https://www.nikkei.com/article/DGXMZO58728450R00C20A5L60000/. (2020).
32. Nihon Keizai Shimbun. Ibaraki Prefecture begins patrolling restaurants to prevent new corona infection. https://www.nikkei.com/article/DGXZQOCC14D990U1A410C2000000/. (2021).
33. Hojokin Portal. Ibaraki Prefecture: Ibaraki Amabie-chan Business Registration Cooperation Fund. https://hojyokin-portal.jp/subsidies/7970. (2020).
34. Hojokin Portal. Ibaraki Prefecture, Mito City: Delivery Service Emergency Relief Fund. https://hojyokin-portal.jp/subsidies/5293. (2020).
35. Hojokin Portal. Kashima City, Ibaraki Prefecture: Kashima City cooperative fund for countermeasures against new coronavirus infection (restaurant business, lodging business, cab, driving agency, sightseeing bus, travel business). https://hojyokin-portal.jp/subsidies/5306. (2020).
36. Yamanashi Prefecture. Emergency measures in Yamanashi Prefecture to prevent the spread of new coronavirus infection. https://www.pref.yamanashi.jp/koucho/coronavirus/documents/measure_200419.pdf. (2020).
37. Yamanashi Prefecture. FY2020 Governor's press conference. https://www.pref.yamanashi.jp/chiji/kaiken/r2.html. (2020).
38. Yamanashi Prefecture. FY2021 Governor's press conference. https://www.pref.yamanashi.jp/chiji/kaiken/r3.html. (2021).
39. Yamanashi Prefecture. Request to shorten business hours for restaurants, etc. https://www.pref.yamanashi.jp/library_documents/insyokuten2.pdf. (2021).
40. Shizuoka Prefecture. Requests based on Article 24, Paragraph 9 of the Act on Special Measures against Pandemic Influenza, etc. https://www.pref.shizuoka.jp/kinkyu/covid-19-kyuugyouyousei.html. (2020).
41. Shizuoka Prefecture. Message from Governor Kawakatsu to the People of the Prefecture. https://www.pref.shizuoka.jp/kinkyu/covid-19-tijikomennto.html. (2021).
42. Shizuoka Prefecture. Status of response to new coronavirus infections. http://www.pref.shizuoka.jp/bousai/event/documents/shiryou5.pdf. (2020).
43. Tokyo Shimbun. Shizuoka Prefecture asks Fuji City to shorten working hours from April 23, the first time in the prefecture under the special measures law. https://www.tokyo-np.co.jp/article/75871. (2020).
44. Nikkei Shimbun. Shizuoka Prefecture to ask restaurants in Kosai City to shorten business hours. https://www.nikkei.com/article/DGXZQOCC176WW0X10C21A5000000/. (2021).
45. Tochigi Prefecture. Overview of Tochigi Prefecture's emergency measures. https://www.pref.tochigi.lg.jp/e04/documents/20020427kinkyuujitaisochigaiyou.pdf. (2020).

46. Tochigi Prefecture. Overview of Tochigi Prefecture's emergency measures. https://www.pref.tochigi.lg.jp/e04/documents/kinkyujitaisoti05070510.pdf. (2020).
47. Tochigi Prefecture. List of facilities subject to facility use restrictions. https://www.pref.tochigi.lg.jp/e04/documents/20200427taisyousisetsu.pdf. (2020).
48. Tochigi Prefecture. Message from the Governor to the Citizens of the Prefecture and Others in Response to the Extension of the Period of the National Emergency Declaration. https://www.pref.tochigi.lg.jp/e04/r020507chijimesseiji.html. (May 5, 2020).
49. NHK. Tochigi Prefecture to lift all requests for closure, no request to refrain from events, corona measures. https://www3.nhk.or.jp/news/html/20200515/k10012432051000.html. (May 15, 2020).
50. Shimotsuke Shimbun. Tochigi Prefecture declares state of emergency, urges restaurants to shorten hours across the region: governor "It is the last resort." https://www.shimotsuke.co.jp/articles/-/406600. (January 14, 2021).
51. Tochigi Prefecture. Message from the Governor on Preventing the Spread of Infections after Emergency Measures (February 4). https://www.pref.tochigi.lg.jp/e04/20210204chijimesse-ji.html. (2021).
52. Nagano Prefecture. List of FY2020 Governor's Press Conference. https://www.pref.nagano.lg.jp/koho/kensei/koho/chijikaiken/2020/index.html. (2020).
53. Chunichi Shimbun. Nagano Prefecture raises alert level to 5 in Iida City, asks restaurants to shorten hours. https://www.chunichi.co.jp/article/186685. (January 17, 2021).
54. Chunichi Shimbun. Nagano Prefecture asks Matsumoto City to close restaurants and shorten working hours, and Hakuba Village is on "5" alert. https://www.chunichi.co.jp/article/189000. (January 21, 2021).
55. NHK. Nagano Prefecture to ask restaurants and other establishments in Nagano City to shorten hours or close April 2–9. https://www3.nhk.or.jp/news/html/20210331/k10012948491000.html. (March 31, 2021).
56. Gunma Prefecture. Emergency Measures in Gunma Prefecture to Prevent the Spread of New Coronavirus Infection. https://www.pref.gunma.jp/contents/100152402.pdf. (2020).
57. Gunma Prefecture. FY2020 Governor's press conferences. https://www.pref.gunma.jp/chiji/z90g_00188.html. (2020).
58. Gunma Prefecture. FY2021 Governor's press conferences. https://www.pref.gunma.jp/chiji/o7000001.html. (2021).
59. Jomo Shimbun. Gunma Prefecture's own guidelines for lifting the suspension of operations: Four-step decision-making process based on internal and external conditions. https://www.jomo-news.co.jp/articles/-/21226. (May 12, 2020).
60. Jomo Shimbun. Restaurants in five cities in Gunma Prefecture asked to refrain from operating after 10 p.m. https://www.jomo-news.co.jp/articles/-/18894. (December 11, 2021).
61. Ibaraki Prefecture. New Coronavirus Infection Emergency measures in Ibaraki Prefecture. https://www.pref.ibaraki.jp/1saigai/2019-ncov/documents/200417kinkyujitaisochi.pdf. (2020).
62. Ibaraki Prefecture. Changes in the Ibaraki version of Corona Next Stage. https://www.pref.ibaraki.jp/1saigai/2019-ncov/stagesuii.html. (2020–2021).
63. Ibaraki Prefecture. List of Governor's press conferences in 2020 regarding the "novel coronavirus infection." https://www.pref.ibaraki.jp/1saigai/2019-ncov/kaiken2020.html. (2020)
64. Ibaraki Prefecture. List of Governor's press conferences (related to the novel coronavirus infection). https://www.pref.ibaraki.jp/1saigai/2019-ncov/kaiken2.html. (2021).
65. Yamanashi Prefecture. Standards pertaining to measures to prevent infectious diseases (restaurant industry). (2020).
